# A quantum crystallographic approach to short hydrogen bonds†

**DOI:** 10.1039/d1ce00355k

**Published:** 2021-08-13

**Authors:** Lucy K. Saunders, Anuradha R. Pallipurath, Matthias J. Gutmann, Harriott Nowell, Ningjin Zhang, David R. Allan

**Affiliations:** Diamond Light Source, Harwell Science and Innovation Campus Didcot OX11 0DE UK Lucy.Saunders@diamond.ac.uk; School of Chemical and Process Engineering, University of Leeds Leeds LS2 9JT UK A.R.Pallipurath@leeds.ac.uk; Research Complex at Harwell Didcot Oxfordshire OX11 0DE UK; EPSRC Centre for Innovative Manufacturing in Continuous Manufacturing and Advanced Crystallization, University of Strathclyde G1 1RD UK; ISIS Pulsed Muon and Neutron Source, Rutherford Appleton Laboratory, Harwell Oxford Didcot OX11 0QX UK; Chemistry, Faculty of Natural and Environmental Sciences, Highfield Campus, University of Southampton Southampton SO17 1HE UK

## Abstract

In this work we use high-resolution synchrotron X-ray diffraction for electron density mapping, in conjunction with *ab initio* modelling, to study short O—H⋯O and O^+^—H⋯O^−^ hydrogen bonds whose behaviour is known to be tuneable by temperature. The short hydrogen bonds have donor–acceptor distances in the region of 2.45 Å and are formed in substituted urea and organic acid molecular complexes of *N*,*N*′-dimethylurea oxalic acid 2 : 1 (**1**), *N*,*N*-dimethylurea 2,4-dinitrobenzoate 1 : 1 (**2**) and *N*,*N*-dimethylurea 3,5-dinitrobenzoic acid 2 : 2 (**3**). From the combined analyses, these complexes are found to fall within the salt-cocrystal continuum and exhibit short hydrogen bonds that can be characterised as both strong and electrostatic (**1**, **3**) or very strong with a significant covalent contribution (**2**). An additional charge assisted component is found to be important in distinguishing the relatively uncommon O—H⋯O pseudo-covalent interaction from a typical strong hydrogen bond. The electron density is found to be sensitive to the extent of static proton transfer, presenting it as a useful parameter in the study of the salt–cocrystal continuum. From complementary calculated hydrogen atom potentials, we attribute changes in proton position to the molecular environment. Calculated potentials also show zero barrier to proton migration, forming an ‘energy slide’ between the donor and acceptor atoms. The better fundamental understanding of the short hydrogen bond in the ‘zone of fluctuation’ presented in a salt-cocrystal continuum, enabled by studies like this, provide greater insight into their related properties and can have implications in the regulation of pharmaceutical materials.

## Abstract 2

Mapped electron density and *ab initio* modelling reveal how H-atom position and molecular environment tune short hydrogen bond characteristics and properties.

## Graphical abstract

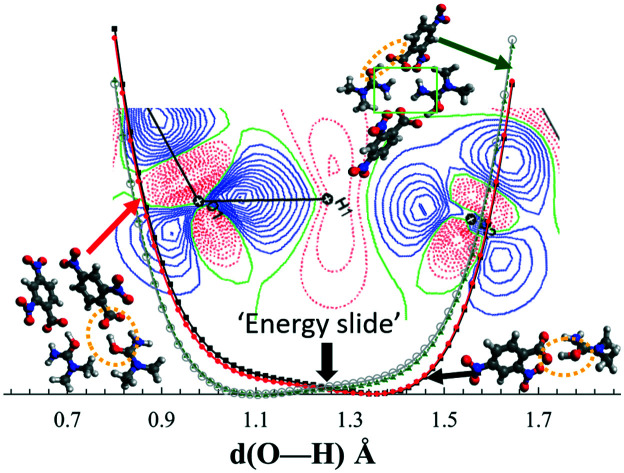

## Introduction

Hydrogen bonds are important in biology and chemistry for their roles in maintaining structure,^1,2^ for molecular recognition^3^ as well as in facilitating reaction pathways.^4,5^ The formation of hydrogen bonds occurs within a set of conditions, as defined by Etter's rules,^6^ where available hydrogen bond donors interact with available acceptors in an order determined by the strength of the resulting interaction. Hydrogen bonds involving oxygen or nitrogen donor/acceptor atoms such as O—H⋯O, N—H⋯O or N—H⋯N tend to be stronger. There is particular interest in the shorter O—H⋯O hydrogen bonds whose donor–acceptor (D–A) distances approach 2.45 Å as they are found to be on the border between strong and very strong interactions.^7^

Specific features of these short, strong hydrogen bonds include a sharing of electron density distribution across the hydrogen bond,^8^ in some cases this may equate to covalency in a 3-centre 2-electron system.^9,10^ The interactions can have hydrogen potentials approaching that of a single well with a diminished barrier to proton transfer^11^ with large values of hydrogen bond energy (>100 kJ mol^−1^).^12,13^ Varying proton positions are often found, either bonded to the original donor (a neutral hydrogen bond), transferred to the acceptor (salt formation) or, in some cases, the donor and acceptor atoms compete for the hydrogen atom such that it may be centred^14^ (salt-cocrystal continuum) or appear on the edge of proton transfer.^15^ Where proton transfer occurs, these are charge assisted hydrogen bonds (CAHBs) and are even stronger due to the additional charges increasing the electrostatic component of the interaction.^16^ Protonation state is particularly important in the regulation of pharmaceutical cocrystals, determining whether the final form is an approved drug or a new chemical species.^17^ Δp*K*_a_ of the interacting components (p*K*_base_ − p*K*_acid_) can be used to predict salt formation by proton transfer, expected if Δp*K*_a_ > 2 or 3.^18^

Temperature dependent proton migration behaviour can be observed across short O—H⋯O hydrogen bonds^19–21^ when formed in organic molecular crystals between acidic and basic molecular components.^22^ The number of systems identified with this behaviour are still relatively few but the proton hopping can have useful applications in ferroelectrics^23^ or colour changing^24^ materials. This behaviour is only observed for systems where the D–A distance is less than 2.45 Å whilst a less negative Δp*K*_a_ (*i.e.* approaching zero) between components may result in larger proton shifts.^19^ Combined inelastic neutron scattering and molecular dynamics simulations also indicate migration across short O—H⋯O hydrogen bonds to be a result of changes in hydrogen atom (H-atom) potentials induced by thermal fluctuations of the molecular environment.^25^

**Fig. 1:**
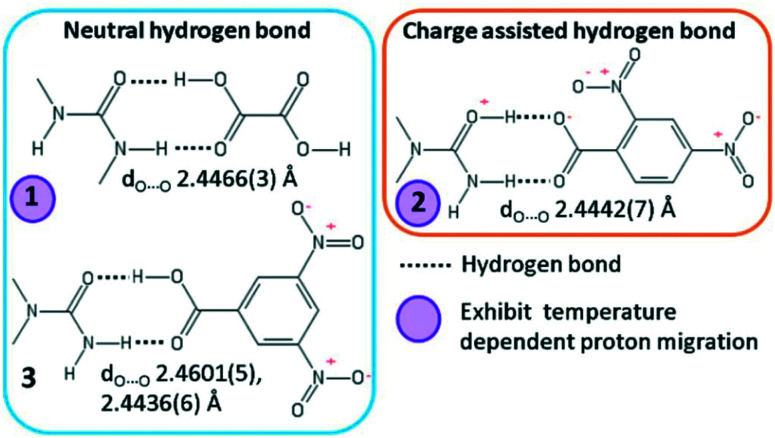
The neutral O—H⋯O and charge assisted O^+^—H⋯O^−^ short hydrogen bonds formed in **1***N*,*N*′-dimethylurea oxalic acid 2 : 1, **2***N*,*N*-dimethlyurea 2,4-dinitrobenzoate 1 : 1 and **3***N*,*N*-dimethlyurea 3,5-dinitrobenzoic acid 2 : 2 (two symmetry independent hydrogen bonded dimers form in the asymmetric unit). The short hydrogen bond proton positions in **1** and **2** are known to be sensitive to temperature, exhibiting temperature-dependent proton migration (highlighted by purple circle).

In this work, we explore short O—H⋯O/O^+^—H⋯O^−^ hydrogen bonds formed in a selection of known substituted urea organic acid cocrystals and salts with O⋯O D–A distances in the region of 2.45 Å, where their character and temperature dependent behaviour are varied (Fig. 1).^19,20^ The short hydrogen bonds in **1** (*N*,*N*′-dimethylurea oxalic acid 1 : 1) and **3** (*N*,*N*-dimethylurea 3,5-dinitrobenzoic acid 2 : 2) are neutral whilst in **2** (*N*,*N*-dimethylurea 2,4-dinitrobenzoate 1 : 1) the hydrogen bond is charge assisted. The proton position is known to be sensitive to temperature in the short hydrogen bonds of **1** and **2**, gradually shifting across the hydrogen bond as a function of temperature (called proton migration).

**Fig. 2:**
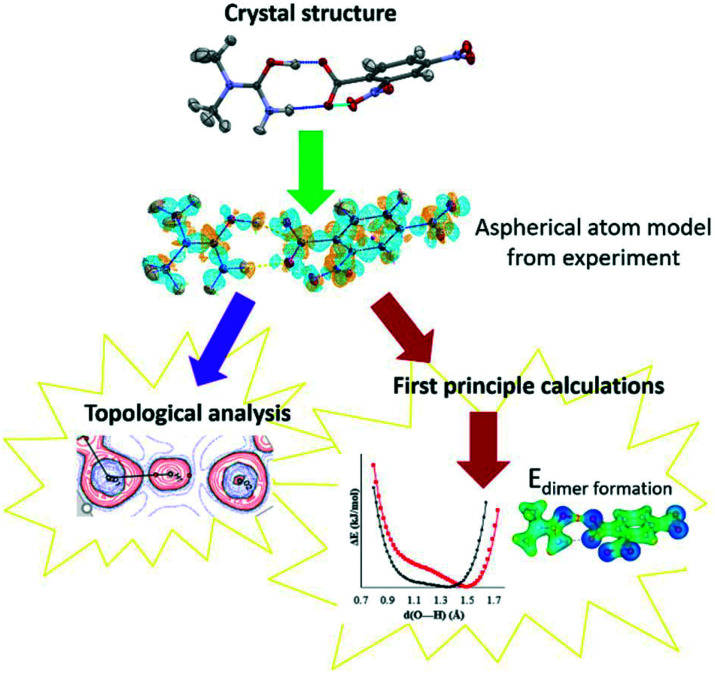
The key components of the combined approach used to study the short O—H⋯O hydrogen bonds of systems **1–3** including topological analysis and first principle calculations. The start point is the aspherical atom model obtained from the multipolar refinement.

Through charge density analysis,^26^ we examine the experimental electron density distribution (EDD) interpreted using Bader's quantum theory of atoms in molecules (QTAIM)^27,28^ from single crystal samples of **1–3**. The experimental EDDs are obtained following a multipolar crystal structure refinement^29^ against high-resolution single crystal synchrotron X-ray diffraction data. Synchrotron X-ray facilities are less routinely used to obtain the EDD despite offering high X-ray flux and tuneable wavelengths.^30^ The experimental EDD analysis is combined with first principle calculations of properties including the reduced density gradient from non-covalent interaction analysis,^31^ the electrostatic potential,^32–34^ PIXEL interaction energies^35,36^ and H-atom potentials.^37–39^ A combined analysis approach is commonly used to assess interaction characteristics in terms of material properties.^40–42^ Here it allows a complete characterisation of the short hydrogen bonds (Fig. 2) so that specific features that determine their properties, such as their differing migration behaviour, can be established.

As this study focuses on hydrogen bonds, additional methods (experimental and computational) are used to improve on the H-atom parameters determined from the X-ray data; typically the X—H distances are underestimated whilst only isotropic displacement parameters for the H-atoms can be refined. Obtaining the best models for the H-atoms is important in charge density analysis where any deficiencies will significantly affect the derived properties involving these atoms (and thus hydrogen bond characterisation).^43–45^ The technique of neutron diffraction allows the most accurate determination of H-atom parameters and has been performed for **2**. In the case of **1** and **3**, the larger single crystals required for neutron diffraction could not be grown and so calculation-based alternatives were implemented on the X-ray structures. Hirshfeld atom refinement (HAR)^46^ with NoSpherA2,^47^ was performed to obtain accurate X—H distances. The SHADE3 server^48^ is a recommended method to obtain ADPs for charge density analysis.^49^ However, in this study, HAR was implemented to obtain the H-atom ADP model, giving the better fit to the experimental data.

## Experimental

The details of the experiments are described in the ESI† and include sample preparation (ESI† 1), diffraction data collection and processing (ESI† 2), crystal structure solution and refinement (ESI† 3) and procedures for the charge density analysis (ESI† 3.4, 4). The implementation of the *ab initio* first principles computational methods is also described (ESI† 5).

### Structure optimisation

Within this study, crystal structures have been optimised either by multipolar refinement for topological analysis of the experimental electron density (ESI† 3.4) or for first principles calculations to obtain complementary properties (ESI† 5). These complementary properties include reduced density gradients from non-covalent interaction (NCI) plotting and analysis^31^ (ESI† 5.3), electrostatic potentials (ESP)^32–34^ (ESI† 5.4), CLP-PIXEL dimer interaction energies^35,36^ (ESI† 5.5) and potentials for H-atom motion (ESI† 5.6). The first principle energetics calculations have been performed on the hydrogen bonded dimers of **1–3** as extracted from the crystal structure following multipolar refinement, ‘as in crystal’, and following geometry optimisation using Gaussian09 (ref. 50) code, B3LYP functional^51^ and 6-31G+(d,p) basis set (ESI† section 5.1).^52^ Calculations are also extended to account for the effects of crystal packing, an important consideration in studies of proton transfer,^53,54^ evaluated using a pseudo-Ewald embedding calculation^55^ and giving a ‘cluster’ structure (ESI† section 5.2). These are discussed in the section describing crystal packing effects.

A gas-phase calculation does not fully account for the deformation of the electron density of the molecule due to the crystal environment, and therefore the conclusions from the modelling must be treated with caution. On the other hand, the use of molecular calculations allows us to use a hybrid functional, a level of theory which is not routinely accessible to solid-state calculations, and it has recently been shown that such hybrid functionals provide a more accurate description of hydrogen bonding.^53^ A particular issue is that B3LYP only accounts partially for dispersive interactions. This would be a particular problem for clusters with π-stacking. We partly mitigated this by keeping the molecule in the crystal structure geometry, as described by our ‘cluster’ structures. We also note that we may expect some errors due to the small 6-31G+(d,p) basis set used. However, this was selected as a balance between cost and accuracy, particularly for the larger cluster models.

## Results

### Structural properties

**Fig. 3:**
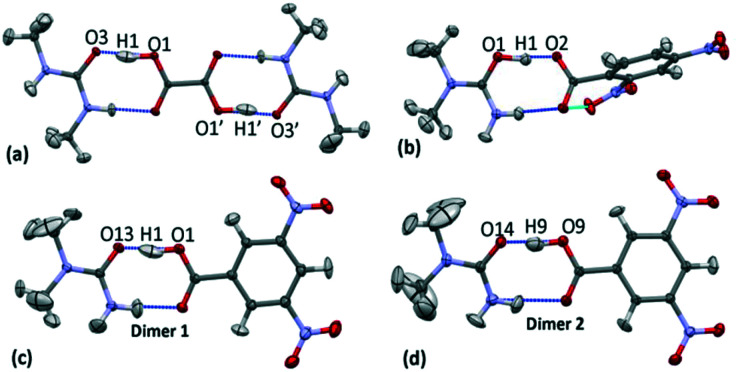
The carboxylic acid : amide R^2^_2_(8) hydrogen bonded dimers in the multipole refined crystal structures with respective H-atom models implemented of (a) **1**, (b) **2**, (c) **3** dimer 1 (**3d1**) and (d) **3** dimer 2 (**3d2**) at 100 K. The oxalic acid molecule in **1** occupies an inversion centre through the central C—C bond and thus the symmetry related portion is shown for completeness.

The crystal structures of **1** (*N*,*N*′-dimethylurea oxalic acid 2 : 1), **2** (*N*,*N*-dimethylurea 2,4-dinitrobenzoate 1 : 1) and **3** (*N*,*N*-dimethylurea 3,5-dinitrobenzoic acid 2 : 2) have a common structural motif of a R^2^_2_(8) carboxylic acid : amide hydrogen bonded dimer formed between substituted urea and organic acid molecular units (Fig. 3).^19,20^ The focus of this study is on the short O—H⋯O hydrogen bonds formed within this motif. In **1–3** the donor–acceptor (D–A) distances of the short hydrogen bonds are *ca.* 2.45 Å (Table 1); the hydrogen bond in **3d1** is slightly longer at 2.46 Å. The O—H⋯O hydrogen bonds are neutral in the case of **1** and **3**; the proton resides on the acid group. The O^+^—H⋯O^−^ hydrogen bond formed in **2** is the only charge assisted interaction of this set; the acidic proton has fully transferred to the *N*,*N*-dimethylurea molecule. Two symmetry independent hydrogen bond dimers form the asymmetric unit of **3**.

**Table 1:** Short O—H⋯O hydrogen bond parameters from the final multipolar refined crystal structure with corresponding HAR or neutron H-atom positions for **1–3**

System	dO⋯O (Å)	dO—H (Å)	dH⋯A (Å)	⁁DHA (°)
**1**	2.4466(3)	1.179(9)	1.280(9)	168(1)
**2**	2.4442(7)	1.16(1)	1.31(1)	170(2)
**3**	d1 2.4602(6)	1.11(1)	1.36(1)	170(1)
	d2 2.4436(6)	1.15(1)	1.31(1)	169(1)

Within the short hydrogen bonds of **1–3**, the O—H bond distances are elongated (from 1.015 Å (ref. 56)) and this feature is more pronounced where the donor–acceptor distances are shorter, in agreement with that found elsewhere.^57^ The short hydrogen bond H-atom (Fig. 3) also has an elongated anisotropic thermal parameter in the case of **1** and **3d1** indicating increased thermal motion of this atom along the hydrogen bond.

### Electron density distribution *ρ*(*r*)

**Fig. 4:**
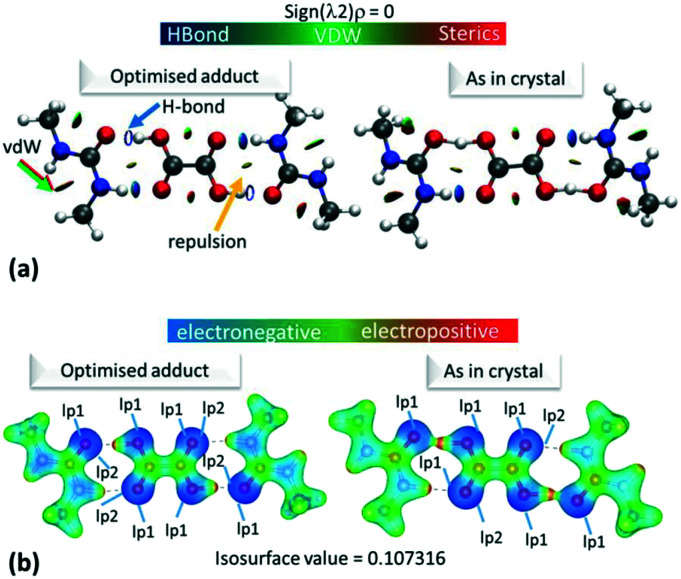
(a) NCI isosurface plot for the hydrogen bonded dimer in **1**. The figure was obtained using a reduced density gradient of 2 a.u. and the blue–green–red values ranging from (−0.05 to +0.05 a.u). Green/red arrows point to regions of vdW interactions, while yellow arrows point to mild repulsions and blue arrows indicate hydrogen bonds. (b) The electrostatic potential surface in **1** calculated for the hydrogen bonded dimer from the optimised or ‘as in crystal’ structure. Red indicates positive regions, blue negative regions and green van der Waals regions. Equivalent plots (a) and (b) for **2** and **3** are located in the ESI,† Fig. S7 and S9.

The electron density distributions (EDD) of the short hydrogen bonds are examined in the topological parameters of the experimentally obtained EDD together with isosurfaces of the calculated reduced density gradient following non covalent interaction (NCI) analysis^31^ (Fig. 4a). The NCI analysis^31^ can be used to directly compare the reduced density gradient (Laplacian ∇^2^(*r*_BCP_)) observed during the multipole reduction of experimental charge densities to those of a comparable molecular adduct in vacuum.^58^ Both analyses are performed at bond critical points of the short hydrogen bonds (BCP; a saddle point in the electron density *ρ*(*r*) where its gradient is ∼0 being a minimum in the direction of the bond and a maximum perpendicular to it).

**Fig. 5:**
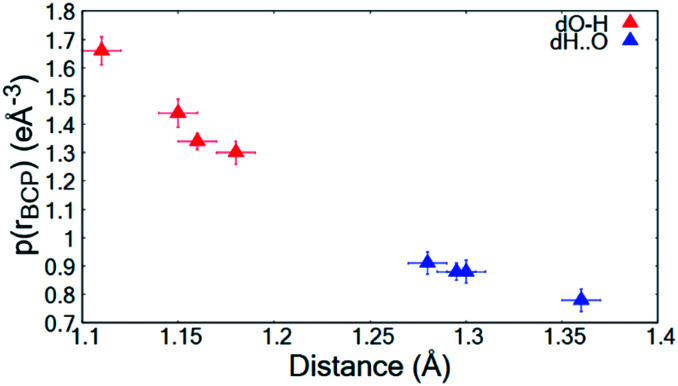
*ρ*(*r*_BCP_) for the O—H and H⋯O BCPs of the short hydrogen bonds in **1–3** as a function of atom–atom separation.

The topological parameters (Table 2) show that the value of *ρ*(*r*)_BCP_ at the O—H/H⋯O BCP decreases with increasing atomic separation (Fig. 5) whilst only a negative value of the Laplacian ∇^2^(*r*_BCP_) for the H⋯O interaction is found in **2** (−1.71(11) e Å^−5^) where full proton transfer has occurred across the hydrogen bond. In agreement with previous findings,^9^ as the electron density reflects proton position in **1–3**, it therefore appears sensitive to the extent of static transfer of the H-atom across the hydrogen bond. The large values of the electron density in the H⋯O region of the short hydrogen bonds given by the topological parameter *ρ*(*r*)_BCP_ (0.79 to 0.9 e Å^−3^) combined with the ring shaped deep blue/covalent bond of the respective calculated NCI isosurface (Fig. 4b and S7†), which exceeds the mapping limits, suggest the short O—H⋯O hydrogen bonding interactions to be strong^59^ and approaching a 3-centre, 2-electron system (*ρ*(*r*)_BCP_ ≈ 1 e Å^3^).^10^ These observations agree with the short hydrogen bond O⋯O separation distance of *ca.* 2.45 Å often being associated with stronger hydrogen bonds. As an indicator of strength, the values of *ρ*(*r*_BCP_) correspond to *ca.* 30% of that determined in the covalent bond of O—H in H_2_O (*ρ*(*r*_BCP_) 2.63 e Å^−3^).^27,60^ In weak to moderate strength hydrogen bonds, typical values are significantly lower on the order of 0.05 to 0.23 e Å^−3^ (ref. 57) and have lighter blue to green calculated NCI isosurfaces, as can be seen for the complementary N—H⋯O interaction in the dimer motifs (Fig. 4a). This analysis shows that both the NCI isosurface and *ρ*(*r*)_BCP_ are useful in assessing hydrogen bond strength whilst *ρ*(*r*)_BCP_ gives a better indicator of position (and can be used as a scaling tool) within the salt–cocrystal continuum.

**Table 2:** Selected topological parameters of the short O—H⋯O/O^+^—H⋯O^−^ hydrogen bond H⋯O BCPs for each system. The energy densities (kinetic *G*_BCP_, potential *V*_BCP_ and total *H*_BCP_) are in units of Hartrees Å^−3^. See ESI† (Table S5) for full list of topological parameters including those of the O—H bond

System	Interaction	*R*_ij_ (Å)	*ρ*(*r*_BCP_) (e Å^−3^)	∇^2^*ρ*(*r*_BCP_) (e Å^−5^)	*G*(*r*_BCP_)	*V*(*r*_BCP_)	*H*(*r*_BCP_)	|*V*|/*G*
**1**	O1—H1⋯O3	1.2806	0.91(4)	3.0(2)	0.82	−1.43	−0.61	1.74
**2**	O1—H1⋯O2	1.2953	0.88(3)	−1.7(1)	0.58	−1.27	−0.7	2.19
**3d1**	O1—H1⋯O13	1.3609	0.78(4)	0.95(1)	0.58	−1.09	−0.51	1.87
**3d2**	O9—H9⋯O14	1.3049	0.88(4)	1.89(2)	0.74	−1.35	−0.61	1.82

A comparison of the values of the topological parameters of the Laplacian ∇^2^(*r*_BCP_) and energy densities (*G*_BCP_, *V*_BCP_, *H*_BCP_) across the systems (Table 2) identify a difference in **2**. For this short hydrogen bond, the ∇^2^(*r*_BCP_) is negative (−1.71(11) e Å^−5^) indicating a concentration of charge in the H⋯O region and, when found alongside a negative total energy density (*H*_BCP_), is characteristic of a ‘very strong’ hydrogen bond with a shared-shell covalent character. In contrast, the values of the Laplacian are positive (∇^2^(*r*_BCP_) > 0) for **1** and **3** corresponding to a depletion of charge within the H⋯O atom–atom region and is characteristic of a closed shell electrostatic interaction or ionic type bonding.^61^ The ratio of the Lagrangian kinetic and potential energy densities (|*V*_BCP_|/*G*_BCP_),^62^ which can be used to further define the hydrogen bonding interactions at the short hydrogen bond BCPs (Table 2), additionally suggest a covalent character for **2**, where the ratio is greater than 2 and in the region attributed to covalent bonds. In the case of systems **1** and **3**, a partial covalency is suggested by the |*V*_BCP_|/*G*_BCP_ ratio, where it lies between 1 and 2 in the intermediate region between electrostatic and covalent. Systems **1–3** therefore add to the cases of short hydrogen bonds that are known to exist in this in-between region, with both electrostatic and covalent contributions.^63^ Short O—H⋯O strong hydrogen bonds formed in related substituted urea organic acid co-crystals and salts are found to have either covalent (urea phosphoric acid 1 : 1 (UPA))^64^ or partial covalent character (urea oxalic acid)^65^ from similar experimental electron density analysis. Of these, UPA^64^ is a system which also exhibits temperature dependent proton migration.^21^ Across the systems exhibiting this type of behaviour (UPA, systems **1** and **2**), there does not appear to be any correlation to the electron density distribution across the short hydrogen bond and the migration behaviour observed. Therefore, despite the electron density being sensitive to the extent of static proton transfer, it does not appear to be useful in predicting the likelihood of further proton transfer events as a function of temperature.

**Fig. 6:**
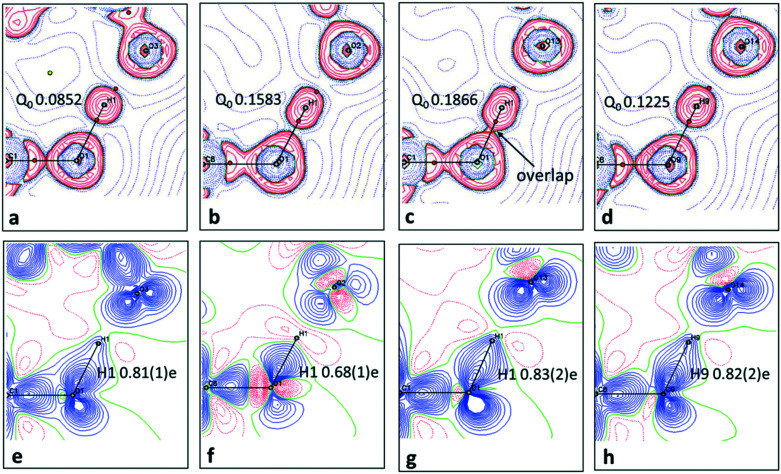
The short hydrogen bonds of **1–3** are visualised in the plane of the O—H⋯O SSHBs in each system. In (a–d), in Laplacian maps showing regions of charge concentration (red contours) or depletion (blue dashed lines) over the interacting groups. The (3, −1) bond critical points are shown as red spheres and importantly show the character of the H⋯O BCP whether located in a charge concentrated or depleted area. Contour lines are at 0.05 e Å^−5^. The refined value of the bond directed quadrupole (*Q*_0_) of the hydrogen bond H-atom is additionally included. In (e–h) static deformation densities. Blue and red colours indicate positive and negative regions of electron density, respectively and reveal lone pairs and bonding density. Contours are drawn at intervals of 0.05 e Å^−3^. The populations for the H-atom, modelled and refined as a monopole, are additionally included in e–h.

When analysing the topological parameters at the H⋯O BCP, the O—H bond directed quadrupole (*Q*_0_) was found to be important in influencing the value of the Laplacian ∇^2^(*r*_BCP_). A larger *Q*_0_ elongates the charge concentration of the H-atom along the interaction increasing likelihood of overlap with the H⋯O BCP and therefore affecting its value. This effect is made clear in the 2D Laplacian maps (Fig. 6a–d). When trialling different ADP models for **1**, they were found to ‘tune’ *Q*_0_ (a less elongated ADP increased the value of *Q*_0_). The importance of obtaining the ‘best’ ADP model for H-atoms in short hydrogen bonds is therefore highlighted, as extracted parameters at the BCPs such as the Laplacian can vary significantly.

### Atomic charges

2D deformation density maps obtained from the experimental electron density distribution (EDD) and showing bonding and lone pair features (Fig. 6e–h) indicate that there is no evidence of charge transfer across the short hydrogen bonds. A connection of the O—H deformation lobes with the acceptor oxygen would be observed in such a case.^66^ This is found to occur across an O—H⋯O hydrogen bond in an identical dimer motif formed in a 1 : 1 urea oxalic acid system.^65^ The nature of the short hydrogen bond is again shown to be different for **2** where, in contrast to **1** and **3**, the H-atom (H1) occupies a negative (red) contour region with a correspondingly low population (0.68(1)e), modelled and refined as a monopole, and has a large, positive net charge (+0.32(1)e) (Table S7†). This evidence points to a deshielded H-atom^57^ significantly depleted of electrons. The large, net positive charge is as found for H-atoms in other charge assisted [O⋯H⋯O]^−^ hydrogen bonds^67^ and is evidence of being involved in the formation of a stronger hydrogen bond, as corroborated by the topological parameters. In **1** and **3** there is no evidence of deshielding of the hydrogen bond H-atom in the deformation density maps, the H-atom position is overlapped by the bonding density, whilst its monopole populations (0.8e) and net atomic charges (*ca.* +0.2e) are correspondingly larger and less positive, respectively.

Across all the short hydrogen bonds, the low monopole population for the H-atom (<1.0e) indicates an extent of charge sharing to the oxygen atom in the O—H bond. The electrostatic potentials for **1–3**, calculated using Gaussian09 (ref. 50) (ESI† 5.4), for the ‘as in crystal’ and optimised hydrogen bonded dimer (Fig. 4b and S9†), visually reflect this reduced population where the H-atom has a positive (red region) ESP in a highly polarised O—H bond (the oxygen opposite the hydrogen in the bond is correspondingly dark blue).

Gaussian09 (ref. 50) calculates the electron density as present in Gaussian orbitals and hence, the ESP diagrams enable the location and contribution of the lone pair (lp) of electrons to the different interactions. As expected, in each case, one of the lp of electrons on the carboxylic acid O(H) is delocalised into the O—H bond while it is easy to spot the location of the other O(H) lp as an increase in electron density, *i.e.* deeper blue in the ESP (Fig. 4b). For the optimised dimer ESP, both oxygen acceptor lps can be located and the lp involved in the hydrogen bond formation sits opposite the positive local potential (red region) on the H-atom. This complementarity generates the idea of a purely electrostatic type interaction for the optimised dimer. However, in the case of the ‘as in crystal’ ESP only one of the lps on the urea O can be located, while the other appears to be diffused over the short hydrogen bond. In contrast to the deformation density maps, the ESP therefore suggest an extent of charge transfer across the short O—H⋯O hydrogen bonds. This effect is reduced in **3d1**, potentially the longer donor–acceptor distance for the short hydrogen bond inhibits the extent of charge redistribution/sharing. This remains the case also in ‘cluster’ structures (Fig. S10 and S11†), but where there is a general delocalisation of electron density over the molecules. The ‘as in crystal’ dimer is as extracted from the crystal structure and so it appears that the sharing of ESP across the components is an element of the crystal packing. The comparison of the optimised *versus* ‘as in crystal’ ESP therefore gives a clear representation of the crystal packing effects on molecular charge transfer.

### Pixel interaction energies

Total (Pixel) interaction energies^68^ have been calculated using the CLP-PIXEL method^36,69,70^ (ESI† 5.5) for each ‘as in crystal’ hydrogen bonded dimer including their decomposition into contributing terms (Table 3). They define the energy of the dimer interaction allowing further distinction in terms of hydrogen bond characteristics.^71,72^

**Table 3:** Decomposition of the total dimer interaction energy (*E*_TOTAL_) into contributing components from CLP-PIXEL calculations for the hydrogen bond dimers in **1–3**. Energies are in kJ mol^−1^

System	*E*_COULOMBIC_	*E*_POLARISATION_	*E*_DISPERSION_	*E*_REPULSION_	*E*_TOTAL_
**1**	−227.3	−158.2	−25.7	350.0	−61.1
**2**	−545.8	−209.1	−24.7	287.0	−492.7
**3d1**	−188.8	−123.3	−23.7	258.1	−77.8
**3d2**	−214.2	−150.6	−24.3	319.8	−69.3

As by the other analyses, the dimer in system **2** is highlighted as being different having a total dimer interaction energy (*E*_TOTAL_) of −493 kJ mol^−1^*versus* those for **1** and **3** that are in the region of −60 to −80 kJ mol^−1^. The *E*_TOTAL_ is significantly larger for **2**; to compare, an O—H covalent bond has a bond energy of *ca.* 465 kJ mol^−1^.^73^ However, this is not unusual for interactions between charged species, thought to occupy the strongly stabilising, ionic coulombic contact zone range.^74^ Using the Hibbert and Emsley classification,^75,76^ the hydrogen bonding interactions forming the dimers in **1** and **3** are considered strong (*E*_TOT_ of −60 to −80 kJ mol^−1^) whilst they are very strong in **2** (*E*_TOTAL_ is −493 kJ mol^−1^). The co-crystal/salt nature of **1–3** is further reflected in the dimer interaction energies where the salt has a significantly larger total interaction energy.

The decomposition of *E*_TOTAL_ into its contributing terms (attractive *E*_COULOMBIC_, *E*_POLARISATION_, *E*_DISPERSION_ and repulsive *E*_REPULSION_) shows that, for all dimers, the coloumbic component contributes most to the stabilisation of the dimer, where *E*_COULOMBIC_ is the largest negative energy. *E*_COULOMBIC_ defines the electrostatic interaction in terms of Coulomb interactions between charges at points ‘pixels’ in space and is not always found to be the dominating term for hydrogen bonds.^77^ The large repulsive contribution (*E*_REPULSION_) could be due to unfavourable secondary diagonal interactions^78^ in the dimer motif between the diagonally opposite carbonyl groups (in the urea and acid) oxygen lone pairs or H-atoms. These repulsive interactions are shown to exist by the yellow NCI isosurfaces (Fig. 4a and S7†) in the centre of the hydrogen bonded ring.

### H-atom potentials

Potential energy surface calculations were performed for the hydrogen bonded dimer with respect to the proton position between the interacting O⋯O atoms in the short hydrogen bonds for **1–3**. This allowed H-atom potentials to be generated. The potentials were produced using Gaussian09 (ref. 50) code and B3LYP functional and 6-31G+(d,p) basis set (ESI† section 5.6) for the ‘as in crystal’ dimer and following its geometry optimisation.

A clear difference is observable between the potentials calculated for the ‘optimised’ *versus* the ‘as in crystal’ dimers (Fig. S12†). Both have the shape of an asymmetric single well potential however, a very small barrier is present in the ‘optimised’ potential which is absent for the ‘as in crystal’ dimer. Optimisation of the dimer fragment also results in a shift in the minimum position, for **1–3**, towards the acid group whilst the O⋯O distance is altered by approximately 0.005 Å. In general, DFT calculations are known to underestimate the strength of short hydrogen bonds resulting in shorter calculated O—H distances and longer calculated O⋯O distances.^25^ This may explain the presence of the ‘shoulder’ like barrier in the optimised potential, where the donor–acceptor atoms are slightly further apart such that well overlap is reduced and barrier height is increased.

**Fig. 7:**
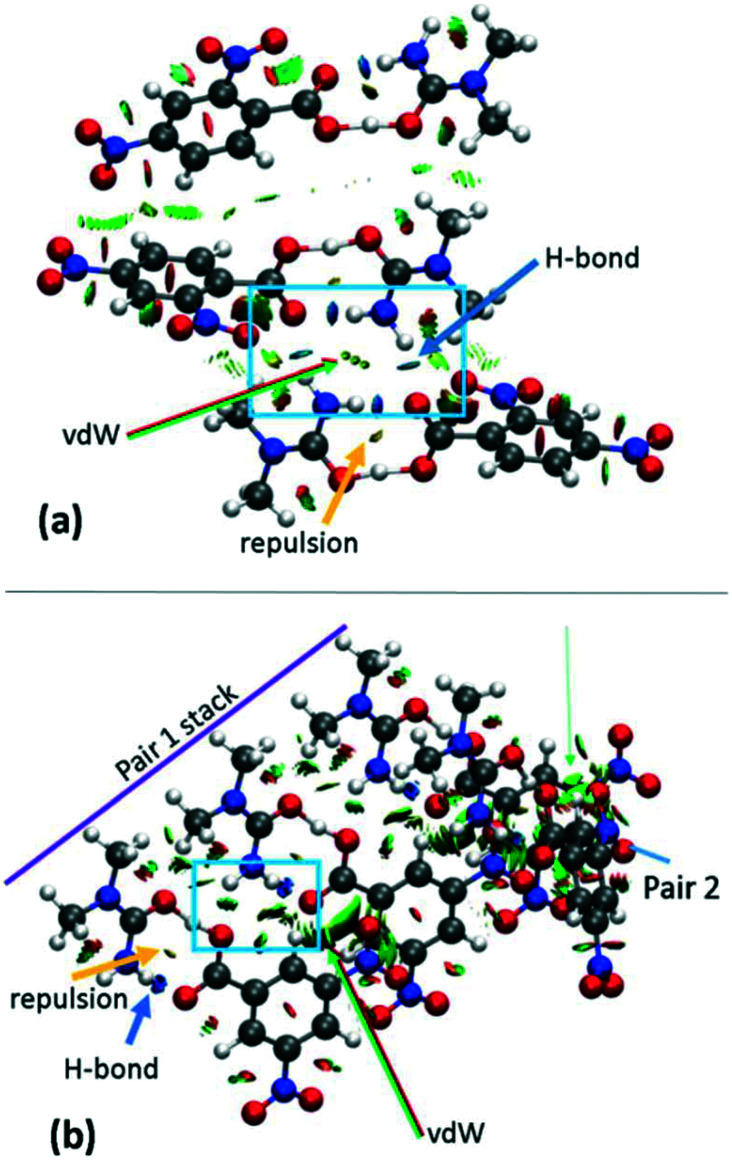
NCI isosurface plot for the cluster models of (a) **2** and (b) **3d1**. The figure was obtained using a reduced density gradient of 2 a.u. and the blue–green–red values ranging from (−0.05 to +0.05 a.u). Green arrows point to regions of vdW interactions, while yellow and blue arrows point to mild repulsions in the middle of hydrogen bonded rings and strong hydrogen bonds, respectively. Equivalent plots for **1** and **3d2** are located in the ESI,† Fig. S8.

The effects of crystal packing are also absent in the optimised *in vacuo* dimer. While the ‘as in crystal’ model does not fully reproduce the bulk crystal environment, it is well known that molecules undergo conformational adjustments on crystal packing^79^ and therefore the difference between the gas-phase ‘as in crystal’ and ‘optimised’ models is not surprising. It is important, however, to consider these effects as they can significantly influence structure,^40^ especially proton transfer state.^80–82^ Furthermore, NCI analysis shows that there are significant weak and strong interactions in the hydrogen bond dimer region (Fig. 7 and S8†) and so should be considered. Therefore, for a truer picture of the H-atom potentials, only the ‘as in crystal’ potentials are considered in the following analysis.

#### ‘as in crystal’ potentials

The asymmetric ‘as in crystal’ potentials (Fig. S12†) indicate unsymmetrical short hydrogen bonds and therefore an asymmetric molecular environment,^10^ as might be expected for a hetero hydrogen bonded dimer. In the absence of a barrier to a second site, the potentials are ‘slide like’ with no clear second minimum. Such a behaviour is also observed from experimental XPS^37^ and molecular dynamics studies^83^ of 3,5-pyridinedicarboxylic acid and urea phosphoric acid^25^ reported in the literature. We also see this for related materials containing the same R^2^_2_(8) carboxylic acid : amide hydrogen bonded dimer (Fig. S13†).

Following on from the other analyses, differences are seen in the case of **2** where the ‘slide’ is significantly shallower such that the potential is closest to resembling a single well flat-bottomed potential; one site is only just favoured energetically over the other. This flatter potential is characteristic of a hydrogen bond with added strength,^11^ as seen from the electron density analysis and Pixel interaction energies. Furthermore, the flatter potential may explain the greater extent of migration observed for this system, as a function of temperature, of the H-atom across the hydrogen bond from the donor to acceptor site. The flatter potential favours the occupation of more H-atom sites along the hydrogen bond. In contrast, for other migration material **1**, the H-atom potential is less flat and a reduced extent of migration is observed; the H-atom undergoes a small shift migrating back towards the acid as a function of temperature rather than across the hydrogen bond.

The donor–acceptor distances for the short hydrogen bonds are similar (*ca.* 2.45 Å) and therefore not likely to be the cause for the flatter potential in **2**. Instead, it could be the added charge assisted component of this interaction, seen in this study to affect other properties of the hydrogen bond. p*K*_a_ values are known to determine the energy barrier height in potentials for moving a H-atom between donor–acceptor wells.^19,84^ For systems **1** to **3**, this does not seem to be the case where the Δp*K*_a_ of components are similar (Δp*K*_a_**1**: −1.94, **2**: −1.74 and **3**: −1.81) whilst the ‘slide’ in the potentials occupy different regions for **1** and **3** (30–40 kJ mol^−1^) *versus* in **2** (*ca.* 10 kJ mol^−1^). One study also formed a link between the extent of temperature dependent migration and Δp*K*_a_.^19^ As was found by Jones *et al.* (2012), the least negative (closer to zero) Δp*K*_a_ in **2** results in the greatest extent of migration observed. However, the trend is not reflected in **3**, where the short hydrogen bonds show no evidence of temperature dependent proton migration yet have a less negative Δp*K*_a_ than **1**, which is a migration material. If taken from the literature, Δp*K*_a_ values should be used with caution as they are often determined for the solution state and are affected by intermolecular interactions in a crystal.^14^ Here they are obtained from the literature^19,85^ and as such are used only as a guide.

#### Crystal packing effects on potentials

The ‘as in crystal’ H-atom potentials are extended to consider nearest neighbours giving ‘cluster’ calculated potentials. Specifically, nearest neighbours are included in differing intermolecular pairs to determine their individual effects on the hydrogen bond dynamics.

**Fig. 8:**
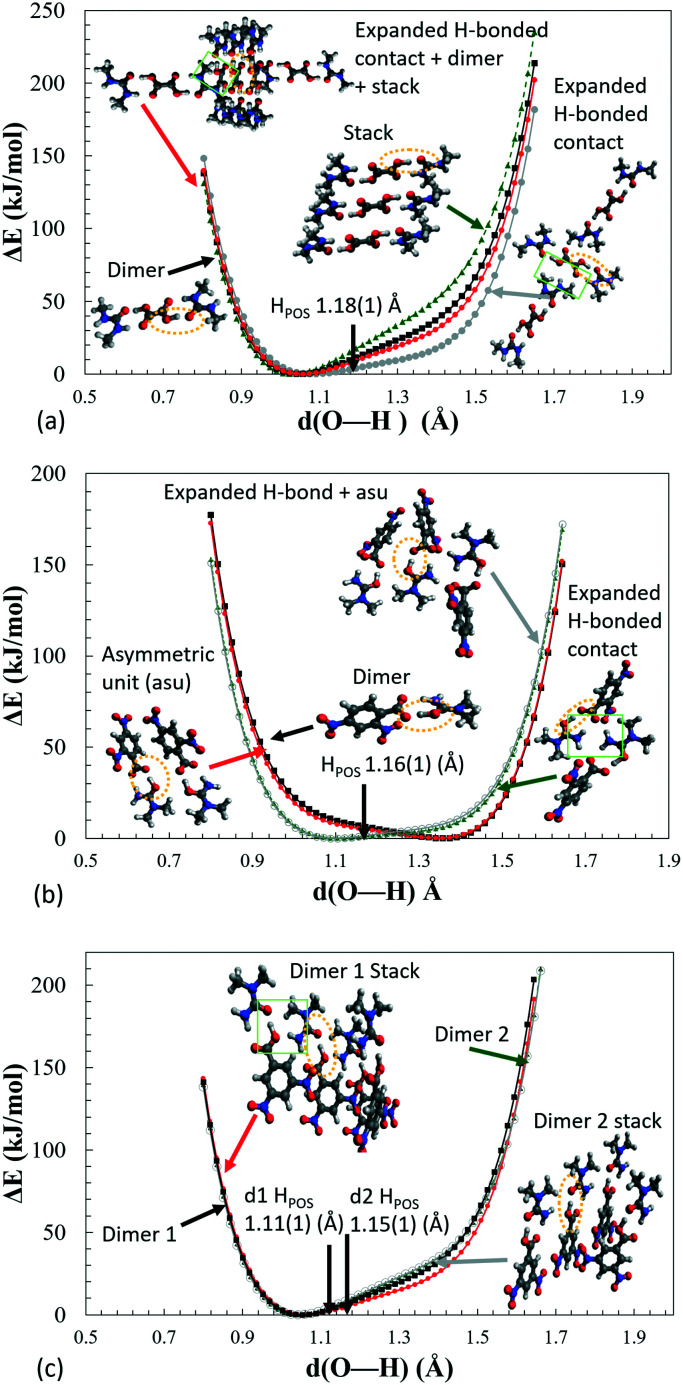
H-Atom potentials *in vacuo* of various ‘cluster’ models of (a) **1**, (b) **2** and (c) **3**, where the proton on the central dimer highlighted by an ellipse (yellow) is moved by 0.02 Å increments between the donor and acceptor oxygens. The green box highlights the presence of extra H-bonding in the expanded H-bonding model. The H-atom position as found in the crystal structure is indicated by black arrows (H_POS_).

Inspection of the extended H-atom potentials (Fig. 8) indicates that, of the intermolecular interactions, the expanded hydrogen bonding contacts cause the most significant changes to the potentials. This might be expected where hydrogen bonding contacts are stronger than π-type interactions and more likely to perturb the local environment. In the most extreme case of **2**, the presence of the hydrogen bonding to the neighbouring dimer unit leads to the inversion of the minima from the acceptor back to the donor, thereby identifying the proton transfer as a feature of these interactions in the crystal environment. In the case of **1** and **3d1**, the extended hydrogen bonding leads to a considerable decrease in the energies of the acceptor and hence in the slope of the PES curve. The local interactions are shown to be weaker in **3d2** by the NCI analysis (ESI† Fig. S8), and there is correspondingly little change between the potential of the dimer alone (asu) *versus* the ‘cluster’ stack. The π-stacking effect leads to an increase in the slope of the potential but in most cases is off-set by the strength of the extended hydrogen bonding with the neighbouring molecules.

As is shown for the extreme case of **2**, by calculating the extended hydrogen bonding cluster potentials for charge assisted hydrogen bonds, if the crystal packing significantly shifts the minima, then this approach may be able to predict whether a material will undergo a complete donor–acceptor temperature dependent proton migration. System **2** could be used as a benchmark for this type of behaviour. This is a hypothesis and other related materials would need to be studied to confirm this.

#### Temperature effects on potentials

The H-atom potential calculations for the ‘as in crystal’ dimers are extended to consider temperature effects for **1** and **2** in order to shed light on their known temperature dependent migration behaviour.^19,20^ Small changes start to occur in the PES at 200 K in **1** and from 250 K in **2**, the ‘slide’ becomes slightly shallower whilst the potential broadens (Fig. S14†). The changes also coincide with a lengthening of the D–A distances of the short hydrogen bonds at these temperatures (Table S9†) and could be the cause of the broadening effect. However, a barrier might be more likely to develop as the donor–acceptor wells separate. Instead, a broadening in the potentials with temperature can be due to thermal fluctuations in the molecular environment, as seen in related migration material of urea phosphoric acid (UPA) 1 : 1.^25^ Furthermore, from molecular dynamics simulations and inelastic neutron scattering^83,86,87^ the mechanism for migration in UPA is attributed to these thermal fluctuations, in particular, low frequency vibrations modes are known to contribute most to the migration occurring.^25,83^

Due to the similarity in the behaviours of the H-atom potentials as a function of temperature for **1**, **2***versus* UPA, and because crystal environment appears to have a significant effect on the H-atom minima positions in the ‘cluster’ structures of **1** and **2**, we propose that their temperature dependent migration behaviour could be due to the same mechanism as UPA, caused by thermal fluctuations in the molecular environment. Furthermore, Boltzmann distributions for **1** and **2**, calculated as a function of temperature, favour this explanation showing a broadening of the population distribution with temperature (greatest in **2**) and not an increasingly populated first excited state (Fig. S15†). The increased population of higher energy levels is the alternative explanation advanced for migration behaviour, as found in other N⋯H⋯O short hydrogen bonded systems.^86,88^ To confirm the nature of the temperature dependent migration behaviour in **1** and **2**, these systems would benefit from further molecular dynamics simulations and inelastic neutron scattering studies. This should be the focus of future work for these systems.

## Conclusions

In this work we have explored short O—H⋯O and O^+^—H⋯O^−^ hydrogen bonds with donor–acceptor distances in the region of 2.45 Å in substituted dimethylurea organic acid salts and co-crystals. The combined approach of experimental charge density analysis and first principles calculations has allowed a complete characterisation of hydrogen bonds within this donor–acceptor distance region.

In the first part we analysed properties of the experimentally determined charge density alongside calculated properties from non-covalent interaction analysis and electrostatic potentials. These properties were also compared to the calculated total Pixel dimer interaction energies. From this, the short hydrogen bonds in *N*,*N*′-dimethylurea oxalic acid 2 : 1 (**1**) and *N*,*N*-dimethylurea 3,5-dinitrobenzoic acid 2 : 2 (**3**) are characterised as strong with a more electrostatic contribution whilst in *N*,*N*-dimethylurea 2,4-dinitrobenzoate 1 : 1 (**2**) the short hydrogen bond is characterised as very strong with a significant covalent contribution. This donor–acceptor distance region therefore remains a zone of fluctuation in terms of the nature of the hydrogen bond. The identification of O—H⋯O hydrogen bonds with a covalent nature is still uncommon. However, a charge assisted component is seen to favour covalency in this interaction and proves to be the determining factor for this set of substituted urea organic acid molecular complexes, in terms of the strength and character of the short hydrogen bonds. Therefore, if salt formation is targeted, this presents a route to access hydrogen bonds with a covalent component.

The relation found between the atom-atom separation or protonation state and the topological properties of the charge density highlights how the electron density in these types of interactions remains highly sensitive to the static proton transfer process. It is therefore a useful parameter in studies of the salt-cocrystal continuum. This has implications for both pharmaceutics and the design of crystalline functional materials. However, for these systems, the electron density distribution does not appear to be useful to predict further proton transfer events as a function of temperature.

In the second part, hydrogen bond characteristics were examined in H-atom potentials, as calculated, and as a function of the crystal packing and temperature. The extensive studies of the H-atom potentials suggest asymmetric hydrogen bonds whose energetics are different when a charge assisted component is included. Extending the potentials to consider crystal packing neighbours, identified by the non-covalent interaction analysis, confirms that proton transfer is an effect of the local environment. Here we further identify that, for these systems, the interactions forming the extended hydrogen bonding have the biggest effect on the H-atom energetics. The similarities in the changes of the potentials as a function of temperature to well-studied migration material urea phosphoric acid suggest that the reported migration of **1** and **2** is likely to also be due to fluctuations in the molecular environment and presents these systems as suitable cases for future inelastic neutron scattering and molecular dynamics studies. System **2** (*N*,*N*-dimethylurea 2,4-dinitrobenzoate 1 : 1) is highlighted as a model system, to add to the well-studied urea phosphoric acid 1 : 1 adduct, for learning about migration materials due to the significant perturbations exhibited by the short hydrogen bond.

Overall, the use of high-resolution synchrotron X-ray diffraction combined with neutron diffraction or Hirshfeld atom refinement has allowed good models of the experimental electron density to be produced in which trends and conclusions about the short hydrogen bonds could be made. Obtaining reliable H-atom models was shown to be important in these studies of short hydrogen bonds, where the H-atom exhibits atypical behaviour as a result of the strength of the hydrogen bond it is involved in. The Hirshfeld atom refinement method, in the absence of neutron data, enabled the study to be extended to additional systems for a better picture of hydrogen bonding characteristics.

## Author contributions

Contributions are defined using CRediT for standardised contribution descriptions. We highlight joint first co-authorship of L. K. Saunders (LKS) and A. R. Pallipurath (ARP) having significant shared data curation and investigation roles with LKS taking lead in conceptualisation of the study and writing the original draft and ARP for writing – review and editing. Credit LKS and ARP for formal analysis, methodology, validation and visualisation. Credit M. J. Gutmann for neutron diffraction data curation and formal analysis. Credit H. Nowell for X-ray diffraction data curation, N. Zhang for software assistance with Gaussian09 and CLP-Pixel calculations and D. R. Allan for providing resources and supervision.

## Conflicts of interest

There are no conflicts to declare.

## Supplementary Material

CE-023-D1CE00355K-s001

CE-023-D1CE00355K-s002
