# Supplementary material for: A quantum crystallographic approach to short hydrogen bonds
Source: CrystEngComm. 2021 Aug 13;23(35):6180–90. doi: 10.1039/d1ce00355k (PMC8436739; doi:10.1039/d1ce00355k)
Supplement: CE-023-D1CE00355K-s001 [file CE-023-D1CE00355K-s001.pdf]

Electronic Supplementary Material (ESI) for CrystEngComm.
This journal is © The Royal Society of Chemistry 2021

Electronic supplementary information

A quantum crystallographic approach to short hydrogen bonds

Lucy K Saunders,*[a] Anuradha R Pallipurath,*[b, c, d] Matthias J Gutmann, [e] Harriott Nowell, [a] Ningjin Zhang, [f] Dave R Allan [a]

[a] Diamond Light Source, Harwell Science and Innovation Campus, Didcot, OX11 0DE, UK.
[b] School of Chemical and Process Engineering, University of Leeds, Leeds, LS2 9JT, UK
[c] Research Complex at Harwell, Didcot, Oxfordshire, OX11 0DE, UK
[d] EPSRC Centre for Innovative Manufacturing in Continuous Manufacturing and Advanced Crystallization, University of Strathclyde, G1 1RD, UK
[e] ISIS Pulsed Muon and Neutron Source, Rutherford Appleton Laboratory, Harwell Oxford, Didcot, OX11 0QX, UK
[f] Chemistry, Faculty of Natural and Environmental Sciences, Highfield Campus, University of Southampton, Southampton, SO17 1HE, UK

Contents

1. Single crystal sample preparation....2
2. Single crystal diffraction data....2
   2.1 High-resolution X-ray diffraction....2
   2.2 Neutron diffraction....3
3. Crystal structure solution and refinement....4
   3.1 Independent Atom Model (IAM)....4
   3.2 Neutron model....6
   3.3 Hirshfeld atom refinement....8
   3.4 Multipolar refinement....9
4. Charge density analysis....14
5. *Ab initio* first principle computational methods....15
   5.1 As in crystal and optimisation....15
   5.2 Influence of neighbouring molecules....15
   5.3 NCI analysis and plotting....15
   5.4 Electrostatic potentials....17
   5.5 CLP-PIXEL....18
   5.6 H-atom potentials....18
   5.7 Boltzmann distributions....21
6. References....22

1. Single crystal sample preparation

All samples were prepared by a method of slow evaporation from solution. Single crystals of *N,N′*-dimethylurea oxalic acid 2:1 (**1**) were grown from an ethanolic solution of the two components (2:1 stoichiometry) at room temperature. Single crystals of *N,N*-dimethylurea 2,4-dinitrobenzoate 1:1 (2) were grown from ethanolic or acetonitrile solutions of the two components (1:1 stoichiometry) at room temperature. Single crystals of *N,N*-dimethylurea 3,5-dinitrobenzoic 2:2 (**3**) were grown from an ethanolic solution of the two components (1:1 stoichiometry) in the fridge at 4 °C. Crystals of this 2:2 *C2/c* form are identified by needle crystals whilst the larger plates indicate the 1:1 $P2_1$ form of this system.[1]

2. Single crystal diffraction data

2.1 High-resolution X-ray diffraction

Single crystal synchrotron X-ray diffraction data were collected on systems **1** – **3** on beamline I19-1[2] at Diamond Light Source, U.K (NR18193) using a Fluid Film Devices Ltd diffractometer equipped with a PILATUS 2M detector. The sample temperature was controlled at 100 K using an Oxford Cryosystems Cryostream Plus. Data were collected at a wavelength of λ = 0.6889 Å. This wavelength was selected to keep the number of 2θ detector positions to a minimum whilst operating at an energy where the efficiency of the beamline Pilatus 2M detector is improved.

To collect the high-resolution data, the detector arm was positioned up to 2θ 80 ° (0.43 Å). At the lower angles, and to ensure good overlap of reciprocal space up to the highest resolution, data were also collected at a 2θ of 0°, 30° and 55°. At each 2θ position, a complete sphere of data were collected (3 x ω, 1 x ϕ scans). At the very low angle, the first set of four runs comprised a single ϕ scan at 2θ = 0° combined with three ω scans at 2θ = 30° and a 0.2°/0.2s oscillation speed. A screening run was first performed at 2θ 0° to determine the optimum level of beam transmission required for each sample. This method implements the inhouse screen19 tool, a programme that provides the user with an assessment of the screen-run X-ray intensities based on maximum resolution of photons and their incidence rate on the detector. For the higher 2θ positions, to ensure accurate high-angle data were obtained, images were collected for longer exposure times using a 0.2°/0.4s oscillation speed whilst the level of beam transmission was increased from the screen19 suggested start value, with this being doubled at 2θ = 55° and quadrupled at 2θ = 80°.

Data collection was performed using the in-house General Data Acquisition (GDA)[3] software and processed using the in-house DIALS[4] for Small Molecule software. The data were additionally scaled and merged using SORTAV[5, 6] software within the WinGX[7] GUI. In SORTAV, an empirical absorption correction was applied based on crystal dimensions and the linear absorption coefficient obtained from the WinGX[7] gui. This method derives an empirical absorption correction for absorption anisotropy by fitting real spherical harmonic functions to the transmission surface.[8] For **2** and **3**, data were truncated at 0.48 Å at the limit of observed diffraction (I/σ<2) (Figure S1, Figure S2). Crystal data are included in Table S1.

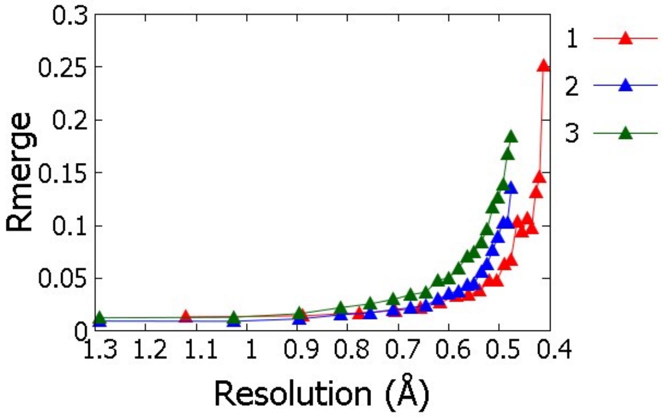

Figure S1 A plot of R_{merge} versus resolution for each dataset of 1 – 3.

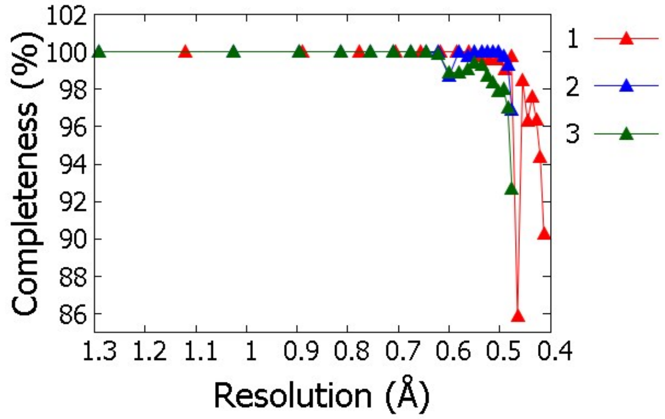

Figure S2 A plot of Completeness (%) versus resolution for each dataset of 1 – 3.

2.2 Neutron diffraction

Only crystals of **2** were suitable for neutron diffraction measurements. A single crystal of *N,N*-dimethylurea 2,4-dinitrobenzoate 1:1 (**2**) was mounted on an Al pin using adhesive Al tape and cooled to 100 K inside a closed-cycle helium refrigerator under He exchange gas. Data were collected at 6 fixed orientations of the crystal on the neutron time-of-flight Laue single crystal diffractometer SXD installed at the ISIS spallation neutron source.[9] The data were processed using the locally available SXD2001 software.[10] The unit cells were taken from X-ray determinations as starting points in the data processing. Crystal data are included in Table S1.

*Table S1 Crystal data for **1 – 3**.*

	**1**	**2**	**2**	**3**
System information	*N,N′*-dimethylurea oxalic acid 2:1	*N,N*-dimethylurea 2,4-dinitrobenzoate 1:1	*N,N*-dimethylurea 2,4-dinitrobenzoate 1:1	*N,N*-dimethylurea 3,5-dinitrobenzoic acid 2:2
Empirical formula	$C_4 H_9 N_2 O_3$	$C_{10} H_{12} N_4 O_7$	$C_{10} H_{12} N_4 O_7$	$C_{20} H_{24} N_8 O_{14}$
Formula weight (g mol^{-1})	133.13	300.24	300.24	600.47
**Crystal Data**				
Source	Synchrotron	Synchrotron	Neutron	Synchrotron
Wavelength (Å)	0.6889	0.6889	0.25 – 8.8	0.6889
Temperature (K)	100	100	100	100
Crystal system	Monoclinic	Orthorhombic	Orthorhombic	Monoclinic
Space group	P 21/n	P b c a	P b c a	C 2/c
a (Å)	3.81790(5)	19.1729(1)	19.157(6)	28.3783(1)
b (Å)	13.2171(2)	5.8157(1)	5.791(2)	6.0988(3)
c (Å)	12.4080(1)	22.9536(1)	22.956(7)	29.7998(1)
α (°)	90	90	90	90
Β (°)	94.6140(10)	90	90	92.511(4)
γ (°)	90	90	90	90
Volume (Å^3)	624.098(13)	2559.41(5)	2546.9(14)	5152.6(3)
Z	4	8	8	8
Density (calculated) (Mg m^{-3})	1.417	1.558	1.566	1.548
Absorption coefficient (mm^{-1})	0.076	0.084	0.351 + 0.002 × λ	0.083
F(000)	284	1248	797	2496
Crystal size(mm^3)	0.057 x 0.030 x 0.028	0.055 x 0.030 x 0.020	3.000 x 1.000 x 1.000	0.055 x 0.052 x 0.025
Θ range (°)	2.186 – 56.505	1.720 – 46.351	10.367 – 86.131	1.326 – 46.348
Reflections collected	9065	12379	4992	24742
Independent reflections	9065 [R(int) = 0.0485]	12379 [R(int) = 0.0467]	4992 [R(int) = n/a]	24742 [R(int) = 0.0457]
Completeness (%) to θ 24.415°	99.8	100.0	Not measurable	100.0

3. Crystal structure solution and refinement

3.1 Independent Atom Model (IAM)

An initial starting model for use in the subsequent refinement techniques (Figure S3) was obtained for **1** – **3** by structure solution using SHELXS[11] and refinement using spherical scattering factors in the independent atom model (IAM). A full-matrix least-squares refinement on F^2 was performed implemented by SHELXL[12] in the WinGX GUI.[7] Non-hydrogen atoms (C N O) were refined with anisotropic thermal displacement parameters. H-atoms were nearly all located from Fourier difference maps and their positions and isotropic thermal parameters allowed to refine freely. Exceptions to this are in **3** where some disorder is indicated in the dimethylurea methyl groups and so the methyl H-atoms for C17 were placed geometrically using the HFIX 137 constraint. Refinement data are given in Table S2.

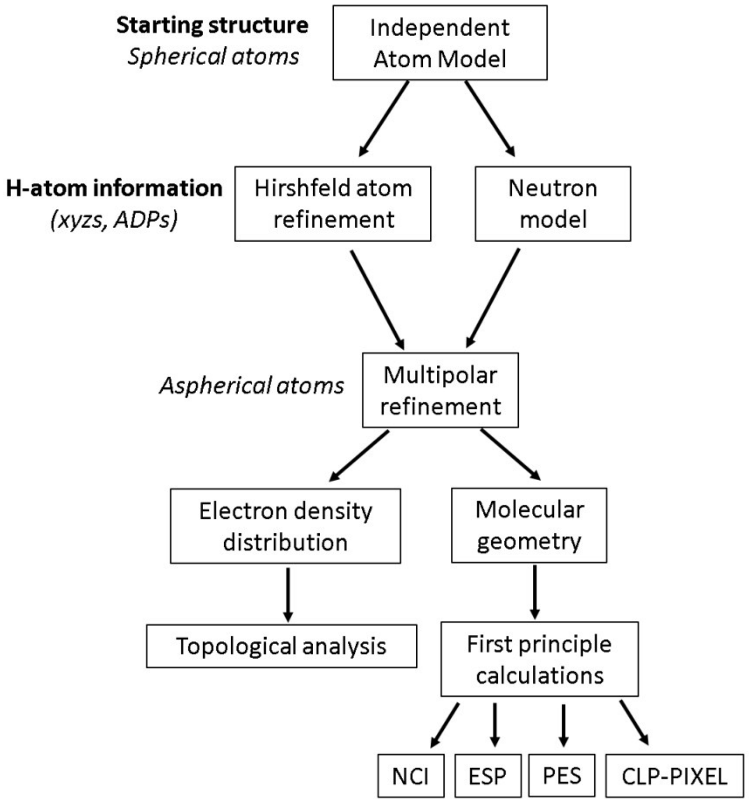

Figure S3 Work flow showing each crystal structure refinement stage, including structural model and H-atom information inputs followed by multipolar refinement outputs for topological analysis or first principle calculations including Non-covalent interaction analysis (NCI), electrostatic potential (ESP), potential energy surface (PES) (H-atom potentials) and dimer interaction energies (CLP-PIXEL).

*Table S2 Refinement data for **1** – **3**. Hirshfeld atom refinement (HAR) details are included only for HAR refined structures **1** and **3** whilst neutron refinement details are included only for neutron refined structure **2**.*

	**1**	**2**	**2**	**3**
System information	*N,N′*-dimethylurea oxalic acid 2:1	*N,N*-dimethylurea 2,4-dinitrobenzoate 1:1	*N,N*-dimethylurea 2,4-dinitrobenzoate 1:1	*N,N*-dimethylurea 3,5-dinitrobenzoic acid 2:2
**Crystal Data**				
Source	Synchrotron	Synchrotron	Neutron	Synchrotron
Temperature (K)	100	100	100	100
**Spherical atom refinement**				
Reflections / restraints / parameters	9065 / 0 / 118	12379 / 0 / 238	4992 / 0 / 304	24742 / 0 / 464
Goodness-of-fit on F^2	1.014	1.022	1.048	1.000
Final R indices [I>2sigma(I)]	R1 = 0.0361, wR2 = 0.1077	R1 = 0.0353, wR2 = 0.1098	R1 = 0.0886 wR2 = 0.2142	R1 = 0.0393, wR2 = 0.1176
R indices (all data)	R1 = 0.0543, wR2 = 0.1157	R1 = 0.0483, wR2 = 0.1160	R1 = 0.0957 wR2 = 0.2274	R1 = 0.0581, wR2 = 0.1271
Extinction coefficient (mm^{-1})	n/a	n/a	0.00049(5)	n/a
ρ_{max}/ρ_{min} (e Å^{-3})	0.581/-0.408	0.601/-0.314	1.462/-1.300	0.502/-0.285
**Hirshfeld atom Refinement (HAR)**				
Reflections /restraints/ parameters	9065/0/163	-	-	24742/0/595
Goodness-of-fit on F^2	0.8748	-	-	0.7239
Final R indices [I>2sigma(I)]	0.0263/0.0616	-	-	0.0279/ 0.0840
R indices (all data)	0.0447/ 0.0678	-	-	0.0466/ 0.0963
ρ_{max}/ρ_{min}/rms (e Å^{-3})	0.3540/-0.3670/ 0.0566	-	-	0.2464/ -0.3705/ 0.0530
**Multipolar refinement**				
Final R(F) indices [I>2sigma(I)]	0.019	0.019	-	0.025
R(F) indices (all data)	0.055	0.039	-	0.054
Final R(F^2) indices [I>2sigma(I)]	0.026	0.019	-	0.025
R(F^2) indices (all data)	0.031	0.0205	-	0.0274
wR(F^2)	0.046	0.042	-	0.053
No of data in refinement	4702	9192	-	17021
Parameters	200	439	-	660
Goodness-of-fit on F^2	1.071	1.009	-	1.028
ρ_{max}/ρ_{min}/rms (e Å^{-3})	0.149/-0.164/0.03	0.199/-0.126/ 0.026	-	0.208/-0.205/ 0.032
Nref/Nv	23.51	20.9385	-	25.7894

3.2 Neutron model

The IAM X-ray model for **2** was used as the initial starting model for the refinement against neutron diffraction data. This insured identical atom naming schemes. Refinement was performed using SHELXL[12] in the WinGX package.[7] The instruction file was updated with the BASF command to

implement relative batch scale factors based on those in the hkl file and to enable wavelength-dependent extinction. The final refinement parameters are in Table S2. The final C, N and O displacement parameters for the neutron and X-ray structures were compared to confirm the need for a scaling of neutron values before implementation into an X-ray model, as is usually performed. In order to implement the neutron H-atom ADPs into the multipolar refinements, they were first scaled to account for these differences using the UIJXN[13] software in the WinGX[7] suite (Table S3 and Table S4). UIJXN[13] compares the anisotropic Uijs from neutron and X-ray diffraction data and outputs scaled neutron Uijs for the H-atoms for use alongside the X-ray model.

*Table S3 Atomic displacement parameters (U_{ij}) in Å^2 for C, N and O atoms in **2**. The first line represents the X-ray values whilst the second line represents the neutron values (grey row).*

**Atom/U_{ij}**	**U11**	**U22**	**U33**	**U23**	**U13**	**U12**
C2	0.01475(11)	0.01744(11)	0.01404(10)	-0.00001(8)	0.00013(8)	0.00049(8)
	0.014(4)	0.007(7)	0.013(4)	0.006(4)	-0.001(3)	0.000(4)
O3	0.02132(12)	0.02790(13)	0.01813(11)	0.00290(9)	0.00576(9)	0.00653(10)
	0.014(5)	0.044(12)	0.015(4)	0.005(6)	0.004(4)	0.005(6)
O2	0.02177(12)	0.02205(11)	0.01875(11)	0.00392(8)	0.00469(9)	0.00604(9)
	0.018(5)	0.034(11)	0.016(5)	0.001(6)	0.006(4)	0.012(6)
N3	0.01686(11)	0.01942(11)	0.01860(11)	0.00207(9)	-0.00222(9)	-0.00403(9)
	0.015(3)	0.024(6)	0.018(3)	0.001(3)	-0.004(2)	-0.004(3)
C7	0.01432(11)	0.01642(11)	0.01498(11)	0.00074(8)	-0.00081(8)	-0.00147(8)
	0.016(4)	0.019(8)	0.013(4)	0.002(5)	-0.001(3)	-0.004(4)
N4	0.02118(13)	0.01792(11)	0.01878(12)	0.00351(9)	0.00078(9)	-0.00076(9)
	0.017(3)	0.023(6)	0.018(3)	0.004(4)	0.003(2)	-0.001(3)
C1	0.01567(11)	0.01970(12)	0.01397(11)	0.00059(9)	0.00081(8)	0.00097(9)
	0.013(4)	0.026(9)	0.011(4)	0.006(5)	0.001(3)	0.000(5)
C5	0.01641(11)	0.01586(11)	0.01598(11)	0.00133(8)	-0.00012(9)	-0.00050(8)
	0.013(4)	0.023(9)	0.015(4)	0.001(5)	0.001(3)	0.003(4)
O4	0.02336(13)	0.02134(12)	0.02105(12)	-0.00235(9)	-0.00322(9)	-0.00461(9)
	0.019(5)	0.036(11)	0.022(6)	0.002(6)	-0.003(4)	-0.005(5)
C6	0.01655(12)	0.01670(11)	0.01477(11)	0.00079(8)	-0.00133(8)	-0.00124(9)
	0.017(4)	0.030(9)	0.013(4)	-0.005(5)	-0.002(3)	-0.003(5)
C3	0.01941(13)	0.01824(12)	0.01595(12)	-0.00136(9)	-0.00200(9)	-0.00136(10)
	0.020(4)	0.020(9)	0.011(4)	0.007(5)	-0.003(3)	-0.002(4)
C4	0.01920(13)	0.01651(11)	0.01862(13)	-0.00042(9)	-0.00169(10)	-0.00208(9)
	0.016(4)	0.028(10)	0.019(5)	-0.001(5)	-0.001(4)	0.000(5)
O6	0.0404(2)	0.02497(14)	0.01655(11)	0.00312(10)	-0.00203(11)	-0.00425(13)
	0.043(7)	0.022(10)	0.015(5)	0.003(6)	0.000(5)	0.003(6)
O7	0.03026(16)	0.02462(14)	0.02918(16)	0.00769(11)	-0.00182(12)	-0.01023(12)
	0.027(6)	0.030(11)	0.024(6)	0.008(7)	0.001(4)	-0.011(6)
O5	0.03592(19)	0.0404(2)	0.02173(14)	0.00721(13)	-0.00120(13)	-0.02093(16)
	0.032(6)	0.037(12)	0.022(6)	0.002(6)	0.002(5)	-0.018(6)
O1	0.01670(10)	0.02709(13)	0.01913(11)	0.00557(9)	0.00229(8)	0.00165(9)
	0.013(5)	0.030(10)	0.016(5)	0.003(6)	0.003(4)	0.004(5)
N2	0.01976(12)	0.02073(12)	0.01513(10)	0.00182(8)	0.00071(9)	0.00107(9)

	0.016(3)	0.032(7)	0.015(3)	0.000(4)	0.002(2)	0.002(3)
C8	0.01616(12)	0.02158(13)	0.01329(11)	0.00103(9)	-0.00039(8)	0.00072(9)
	0.013(4)	0.036(9)	0.010(4)	0.004(5)	0.003(3)	0.000(4)
N1	0.01754(12)	0.02826(15)	0.02225(14)	0.00753(11)	0.00320(10)	0.00466(11)
	0.016(3)	0.021(6)	0.022(4)	0.007(4)	0.003(3)	0.004(3)
C10	0.02258(16)	0.02832(18)	0.01895(14)	0.00437(12)	0.00192(12)	-0.00199(13)
	0.022(5)	0.020(10)	0.019(5)	0.005(6)	-0.001(4)	-0.001(5)
C9	0.02424(17)	0.02470(17)	0.02732(18)	0.00483(13)	0.00041(14)	0.00508(13)
	0.021(5)	0.027(11)	0.020(5)	0.005(6)	0.003(4)	0.006(6)

*Table S4 Atomic displacement parameters (U_{ij}) in Å^2 for H atoms in **2**. The first line represents the un-scaled neutron values whilst the second line represents the UIJXN scaled neutron values (grey row).*

Atom/U_{ij}	U11	U22	U33	U23	U13	U12
H3	0.039(11)	0.09(3)	0.022(11)	-0.010(13)	-0.002(8)	-0.017(13)
	0.03502	0.066734	0.021948	-0.00671	-0.00154	-0.01205
H6	0.029(9)	0.049(19)	0.018(9)	-0.007(11)	-0.005(7)	-0.002(10)
	0.027755	0.038204	0.019392	-0.00488	-0.00383	-0.00488
H4	0.038(11)	0.034(19)	0.038(11)	0.006(12)	0.002(9)	-0.013(11)
	0.033801	0.027262	0.033043	0.004665	0.001404	-0.00983
H1B	0.016(9)	0.10(3)	0.030(11)	0.008(13)	0.012(7)	0.007(11)
	0.0187	0.07333	0.027673	0.006072	0.008021	0.004382
H1A	0.035(11)	0.019(18)	0.033(11)	0.008(11)	0.007(8)	0.004(9)
	0.031976	0.016731	0.029435	0.005529	0.004559	0.002105
H10C	0.065(15)	0.07(3)	0.040(14)	-0.005(16)	0.015(12)	-0.015(15)
	0.052684	0.049697	0.034464	-0.00302	0.009999	-0.01094
H10B	0.060(15)	0.06(3)	0.072(18)	0.03(2)	0.028(12)	-0.004(16)
	0.049034	0.046026	0.056412	0.020755	0.019632	-0.00312
H9B	0.045(16)	0.04(3)	0.13(3)	0.00(2)	-0.009(14)	0.009(16)
	0.03899	0.032347	0.099353	-0.00183	-0.00663	0.005782
H10A	0.034(12)	0.10(3)	0.049(14)	0.031(15)	-0.005(10)	-0.004(14)
	0.031001	0.072634	0.040559	0.022134	-0.00335	-0.00339
H9C	0.056(16)	0.17(5)	0.059(18)	-0.02(2)	-0.028(13)	0.04(2)
	0.046422	0.122275	0.047615	-0.0148	-0.02004	0.027848
H9A	0.050(14)	0.07(3)	0.090(19)	0.052(18)	0.044(13)	0.037(15)
	0.04234	0.052539	0.069068	0.036197	0.030309	0.024958
H1	0.050(14)	0.07(3)	0.090(19)	0.052(18)	0.044(13)	0.037(15)
	0.02827	0.02892	0.02286	0.002012	0.0021	0.007134

3.3 Hirshfeld atom refinement

For **1** and **3** Hirshfeld atom refinement (HAR)[14] was performed on the initial IAM X-ray structures to obtain X—H distances and models for the hydrogen atom anisotropic thermal parameters. HAR was first implemented in the Olex2[15] gui using the NoSpherA2[16] interface under the olex2.refine G-N

option. NoSphera2 extras included using ORCA[17] for wavefunction calculation, the def2-TZVPP basis set alongside the PBE method. The HAR process was iterated at a maximum of 10 cycles to reach convergence. Anisotropic refinement of H-atoms was also selected. The asymmetric unit was used as the initial crystal fragment in **3** whilst in **1**, the oxalic acid molecule occupies an inversion centre in the crystal structure and so was completed for the calculation.

3.4 Multipolar refinement

The experimental electron density distribution of **1** – **3** was obtained from the X-ray diffraction data by implementing aspherical scattering factors in a multipolar refinement model. In this method, crystal structure refinement is expanded from considering atoms as point scatterers to considering their aspherical contribution to the atomic scattering (the multipolar model). This model uses the multipole formalism of Hansen and Coppens,[18] implemented in the XD2006[19] program package using the *XDLSM* refinement routine.

The aspherical electron density is obtained in the multipolar model:

$$\rho(r) = \rho_{core}(r) + P_{val}\kappa^3\rho_{val}(r) + \sum_{l=0}^{l_{max}} \kappa'^3 R_l(\kappa' r) \sum_{m=0}^{l_{max}} P_{lm\pm} y_{lm\pm}(\theta,\varphi)$$

Equation 1

Where the first two terms correspond to the spherically averaged core $\rho_{core}(r)$ and valence $\rho_{val}(r)$ electron densities whilst the third term corresponds to the non-spherical valence density. P_{val} and $P_{lm\pm}$ are the population parameters, κ and κ′ are the expansion/contraction parameters, $y_{lm\pm}$ includes the spherical harmonic angular functions and R_l is the radial function.

The IAM model was used as the start point for **1** – **3**. Refinement was performed on F^2 and using the following steps. Spherical atom refinement was performed initially including (1) the refinement of a scale factor against all data. This was followed by (2) a high order refinement ($\sin\theta/\lambda > 0.6$ Å^{-1}) of non-H-atom positional and thermal parameters and (3) a low order refinement ($\sin\theta/\lambda < 0.6$ Å^{-1}) of H-atom positional and thermal parameters. This allowed optimal atomic coordinates and thermal parameters to be achieved for all atoms. To derive a good starting model for the multipole refinement, (4) starting values for the multipole and κ parameters were imported from the Invariom database[20] using the programme InvarionTool.[21] Each imported Invariom contains theoretically predicted multipole population parameters with relative orientation defined (including local site symmetry and coordinate system) to fit with the starting molecule. These first steps were performed in Linux using a bash script.

In step (5), the H-atom X—H bonds were elongated to distances obtained from either the HAR (**1**, **3**) or neutron refinements (**2**). In the case of **3**, the methyl positional parameters were reset to those from HAR. (6) Anisotropic models for the H-atom thermal parameters were imported into each structure. In the case of **2**, these were from the 100 K neutron structure whilst they were used from HAR in **1** and **3**. The aspherical atom refinement was carried out by (7) a stepwise multipole expansion (M, κ, D, Q, O, H) for all atoms and truncated at the quadrupole level for H-atoms (refining Q0 only) and the hexadecapole level for non H-atoms. All parameters were refined together in the last step (8). Only for **2** were the non-H κ parameters refined. κ′ parameters were kept fixed for all atoms at Invariom database values. For the H-atoms, κ and κ′ were kept fixed at the data base values (ca 1.11 for κ and 1.2 for κ′). The X-ray data used in the refinement were truncated at an appropriate $\sin\theta/\lambda$, determined by considering the agreement between F_{obs} and F_{calc} at the high resolution (**1** 1.0245 Å^{-1}, **2** 1.0245 Å^{-1} and **3** 1.0233 Å^{-1}). The difference mean-square displacement amplitudes (DMSDAs) for all bonds involving non-H atoms were within Hirshfeld limits (< 10^{-4}). Exceptions to this were in **3**,

involving the methyl groups having values at 10 – 14 x 10^{-4}. This may indicate a slight rotational motion of this group, which is not adequately modelled by the ADP tensor; it was not however large enough to be included in the model.

Residuals around several of the oxygen atoms in **2** and **3** indicated an extent of anharmonic motion for these atoms.[22, 23] This was found mainly for nitro group oxygen atoms but was also present on the acid carbonyl oxygen atom in **2**. The anharmonic motion was modelled up to the third order of Gram-Charlier expansion and this improved the residual density around these atoms.

The validity of the multipolar refinement was established by the assessment of the residual density. The residuals in **1** – **3**, visualised in 3D maps using MoleCoolQT,[24] were found to be low whilst systematic features indicative of an incorrect model, such as un-modelled bonding density, or error in the diffraction data, were absent. The fractal dimension plots resulting from the Meindl and Henn[25] analysis (Figure S4a to Figure S6a) gave Gaussian distributions and do not contain any significant features that would suggest an error with either the model or in the diffraction data. Plots of the variation in ratio of $\sum(F_{obs}^2)/\sum(F_{calc}^2)$ versus $sin\theta/\lambda$ (*DRK*-plot)[26, 27] (Figure S4b to Figure S6b) remain within the 5% tolerance required for a satisfactory model, free of significant data problems.[22]

Hirshfeld atom refinement (HAR)[14] ADP models were used over (SHADE3[28]) alternatives in the final multipolar refinements where they resulted in fewer residuals in the vicinity of the short hydrogen bond H-atom position whilst the $\sum(F_{obs}^2)/\sum(F_{calc}^2)$ versus $sin\theta/\lambda$ *DRK*-plot and fractal dimension plots showed fewer deviations from unity or a gaussian shape, respectively.

3.4.1 Topological properties of the electron density distribution

The electron density distribution (EDD) is then examined by extracting its topological properties (the relative arrangement in space)[44] at bond critical points (BCPs)[45-47] between atoms where a bond path exists and where the gradient of the electron density is equal to zero ($\nabla\rho(r) = 0$). Important extracted parameters at BCPs include the electron density ρ(r) and the Laplacian $\nabla^2\rho(r)$ revealing where electronic charge is concentrated or depleted. Other parameters include energy densities, quantifying the energetics of the electron density at the BCP.[36] Net atomic charges and populations of the atoms involved in hydrogen bond formation can also be derived using the QTAIM[33, 34] analysis.

Topological parameters at bond critical points (BCPs) between atoms were extracted from the experimental electron density using the XDPROP module in XD2006.[19] These include $\rho(r)$, $\nabla^2\rho(r)$ and the kinetic ($G(r_{CP})$) and potential $V(r_{CP})$ energy densities, calculated from the electron density using the Abramov approximation:[29]

$$G(r_{CP}) = \frac{3}{10}(3\pi^2)^{\frac{2}{3}}\rho^{\frac{5}{3}}(r_{CP}) + \frac{1}{6}\nabla^2\rho(r_{CP}) \qquad \text{Equation S1}$$

$$V(r_{CP}) = \frac{\hbar^2}{4m}\nabla^2\rho(r_{CP}) - 2G(r_{CP}) \qquad \text{Equation S2}$$

G_{BCP} indicates the mobility of the electrons at the BCP and the pressure they exert on surrounding electrons whilst $V(r_{BCP})$ indicates the pressure exerted on the electrons at the BCP by other electrons.[30] The total energy density $H(r_{CP})$ (the balance between the two) is:

$$H(r_{CP}) = G(r_{CP}) + V(r_{CP}) \qquad Equation\ S3$$

Using XDPROP, BCPs between atoms were searched in rho (ρ) using the CPSEARCH instruction with atom distance limits of 0.7 to 1.7 Å (these limits cover the range of atomic separations from bonds to intermolecular interactions). The topological parameters at each identified BCP were then extracted from the electron density using the molecular graph instruction (MOLGR) in XD.mas instruction file. Monopole populations (P_{VAL}) and net atomic charges were obtained from the XDGEOM module.

All Laplacian and deformation density maps were generated using the XDPROP module and were plotted in MAPVIEW of the XD2006[19] suite.

(a)

(b)

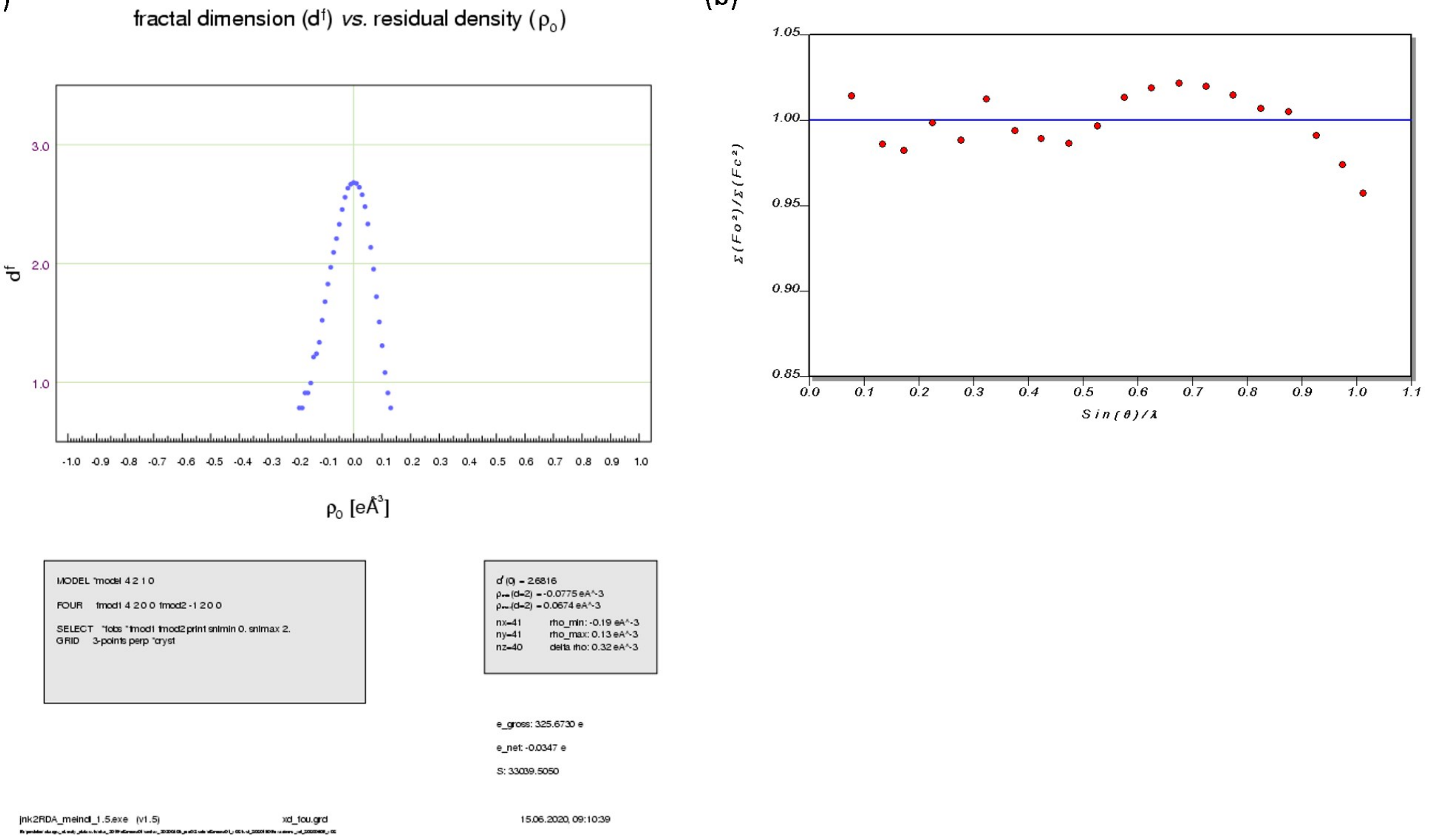

*Figure S4 For system **1**: (a) Fractal dimension analysis of the residual density output using jnk2RDA[25] on the xd_fou.grd for the whole crystal and (b) DRK-plot of* $\sum(F_{obs}^{2})/\sum(F_{calc}^{2})$ *versus* $sin\theta/\lambda$.

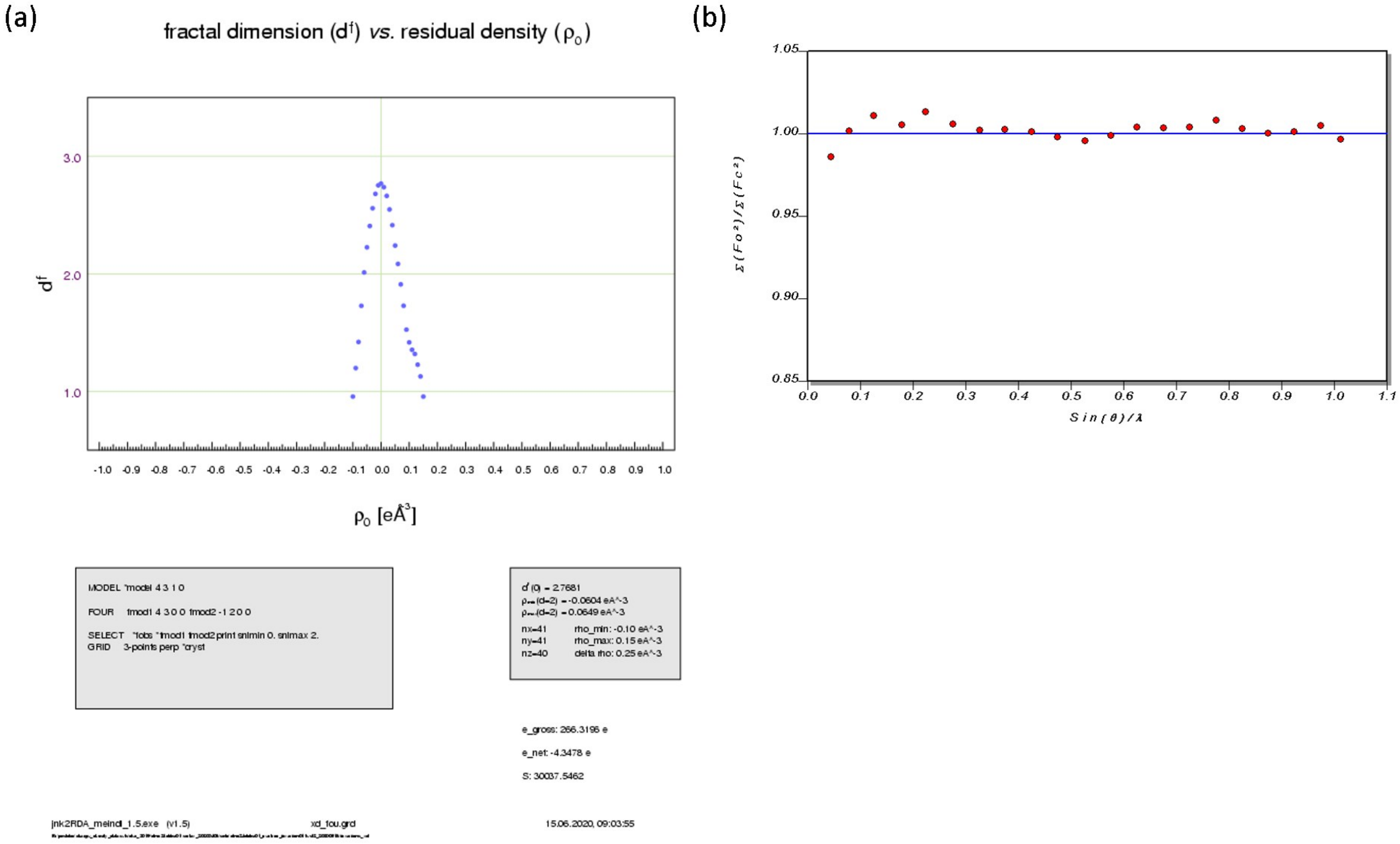

*Figure S5 For system **2**: (a) Fractal dimension analysis of the residual density output using jnk2RDA[25] on the xd_fou.grd for the whole crystal and (b) DRK-plot of* $\sum(F_{obs}^{2})/\sum(F_{calc}^{2})$ *versus* $sin\theta/\lambda$.

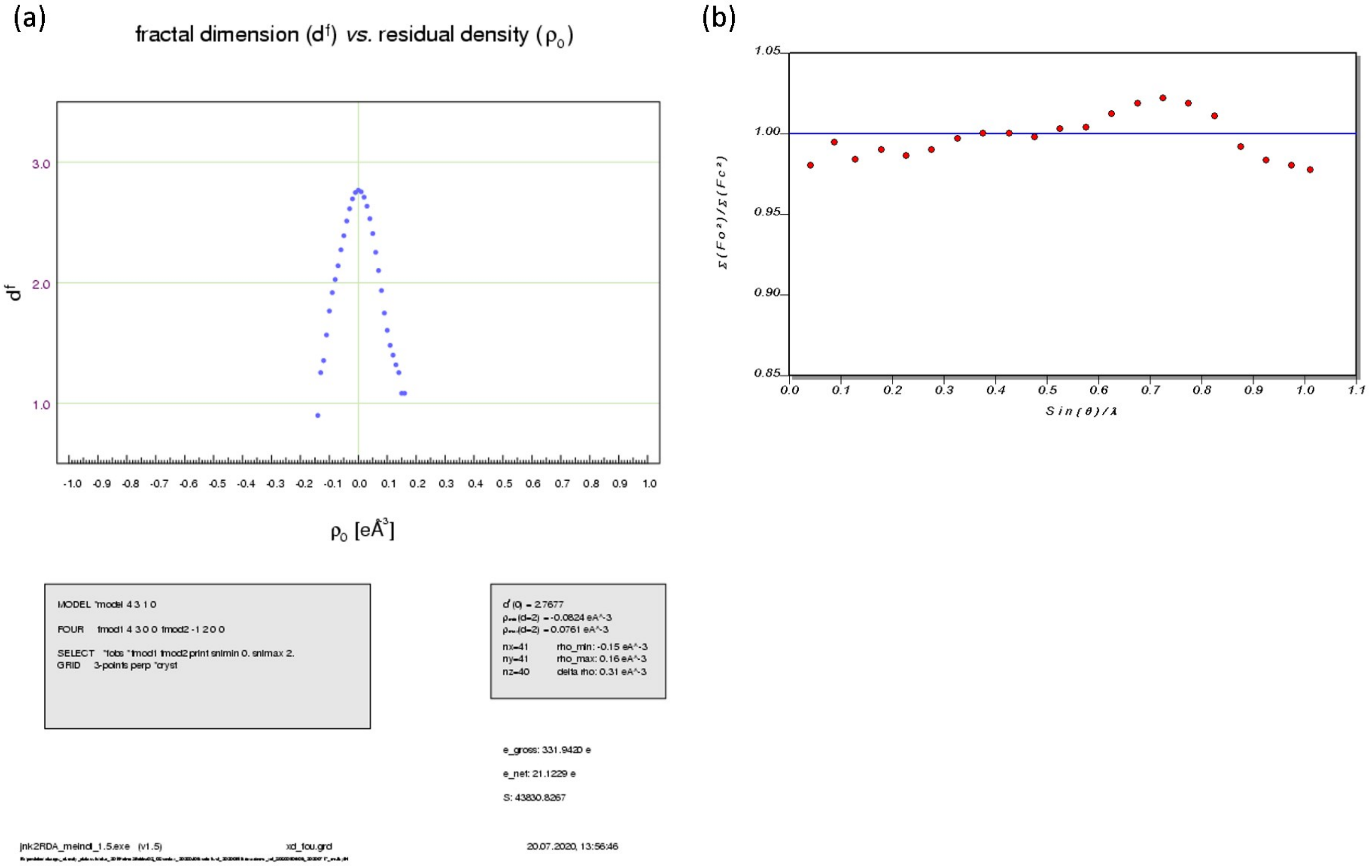

*Figure S6 For system **3**: (a) Fractal dimension analysis of the residual density output using jnk2RDA[25] on the xd_fou.grd for the whole crystal and (b) DRK-plot of* $\sum(F_{obs}^{2})/\sum(F_{calc}^{2})$ *versus* $sin\theta/\lambda$

4. Charge density analysis

*Table S5 Topological analysis of the short O—H···O/ O^+—H···O^- hydrogen bonds for each system **1** - **3**. Energy densities are in units of Hartrees $Å^{-3}$.*

System	Interaction	BCP	Rij d1 (Å)	d1 (Å)	d2 (Å)	ρ(r_{BCP}) $(eÅ^{-3})$	$\nabla^2\rho(r_{BCP})$ $(e\,Å^{-5})$	l1	l2	l3	ε	G (r_{BCP})	V (r_{BCP})	H (r_{BCP})	|V|/G
**1**	O1—H1···O3	O—H	1.179	0.8629	0.3161	1.30(4)	-5.4(2)	-15.03	-14.64	24.31	0.03	1.00	-2.36	-1.37	2.36
		H···O	1.2806	0.9061	0.3745	0.91(4)	3.0(2)	-8.39	-8.34	19.74	0.01	0.82	-1.43	-0.61	1.74
**2**	O1—H1···O2	O—H	1.16	0.8852	0.2748	1.34(3)	-14.4(2)	-18.13	-18.01	21.77	0.01	0.63	-2.27	-1.64	3.60
		H···O	1.2953	0.9418	0.3534	0.88(3)	-1.7(1)	-9.24	-9.06	16.59	0.02	0.58	-1.27	-0.7	2.19
**3d1**	O1—H1···O13	O—H	1.11	0.8313	0.2787	1.66(5)	-19.5(3)	-22.66	-22.54	-22.64	0.01	0.96	-3.29	-2.33	3.43
		H···O	1.3609	0.9479	0.4131	0.78(4)	0.95(1)	-7.34	-7.21	15.5	0.02	0.58	-1.09	-0.51	1.87
**3d2**	O9—H9···O14	O—H	1.15	0.8494	0.3006	1.44(5)	-9.9(3)	-17.67	-17.55	25.34	0.01	1.02	-2.73	-1.71	1.66
		H···O	1.3049	0.9204	0.3844	0.88(4)	1.89(2)	-8.39	-8.17	18.46	0.03	0.74	-1.35	-0.61	1.82

Table S6 Parameters confirming the positive interpenetration of the van der Waals spheres of hydrogen and acceptor atoms at the BCP in the short O—H···O hydrogen bonds. Considered parameters include the bonded radii (r_A, r_H) which are the distances between the atom and the BCP and the nonbonded van der Waals radii (r_A^0, r_H^0). $\Delta r_H = r_H^0 - r_H$ *and* $\Delta r_A = r_A^0 - r_H$.

System	H···A	r_H(Å)	r_A(Å)	r_A^0 (Å)	r_H^0 (Å)	Δr_H (Å)	Δr_A (Å)	$\Delta r_H + \Delta r_A$ (Å)
**1**	H1···O1	0.3745	0.9061	1.52	1.2	0.8255	0.6139	1.4394
**2**	H1···O2	0.3534	0.9418	1.52	1.2	0.8466	0.5782	1.4248
**3d1**	H1···O1	0.413	0.9479	1.52	1.2	0.787	0.5721	1.3591
**3d2**	H9···O9	0.3844	0.9204	1.52	1.2	0.8156	0.5996	1.4152

Table S7 The monopole populations (P_{VAL}) and net atomic charges (Q) obtained from monopole refinement (M) and output from the XDGEOM module using XD2006.[19] Net atomic charges (Q) are defined as the atomic number z minus the sum of the core population, Pcore. [31]

System	Atom	P_{VAL} (e)	Q (e)
**1**	H1	0.81(1)	+0.192(14)
**2**	H1	0.680(11)	+0.320(11)
**3d1**	H1	0.83(2)	+0.17(2)
**3d2**	H9	0.82(2)	+0.18(2)

5. *Ab initio* first principle computational methods

5.1 As in crystal and optimisation

The molecular adducts used for the first principle calculations are the hydrogen bonded dimers in **1** – **3** extracted from the crystal structure following multipolar refinement. The molecular adduct was used either 'as in crystal' or optimised using Gaussian09[32] code, B3LYP functional[33] and 6-31G+(d,p) basis set.[34] Optimisation of the Kohn-Sham orbitals was carried out to the default tolerances of changes in the density matrix, and the geometry to associated default tolerances of the change in forces and displacements of atoms.

5.2 Influence of neighbouring molecules

Calculations were also carried out to identify the effect of interactions in the crystal packing and local to the hydrogen bonded dimer molecular adducts, such as the effect of π-stacking and/or extended hydrogen bonding interactions. Here a pseudo-Ewald embedding type calculation was carried out where the additional molecules and interactions were fixed in place and the proton position of the central dimer was moved manually to generate 51 starting structures for the H-atom potential (potential energy surface) studies, as explained in section 5.5 for a single dimer.

Single point energy calculations, electrostatic potential (ESP) and non-covalent interaction (NCI) analysis for the various expansions of the dimer interactions in the gas phase, to resemble those close interactions in the crystal structure, were carried out. This is in the spirit of the 'Ewald embedding' or 'electrostatic embedding'[35] and these models will be referred to as the 'cluster' structures in the text henceforth. Unlike in 'Ewald embedding' we do not convert these interactions into point charges but merely include them as is, in the structure models used for the following studies. Similar to the 'as in crystal' calculations, the structures were not optimised to prevent changes in both the O···O distances and the relative orientations of groups. A comparison of these with optimised periodic structures could prove useful in studying these interactions holistically, but we consider the current model to be sufficient for the present study. The xyz files with the starting structures are available as SI.

5.3 NCI analysis and plotting

Non-covalent interaction (NCI) analysis[36] of molecular adducts was carried out for **1** – **3** using the Multiwfn code[37] on the .fchk files obtained from optimisation / single point energy calculations using Gaussian09[32] code (B3LYP functional and 6-31G+(d,p) basis set). A high quality grid with 1728000 mesh points was used for these calculations to obtain the reduced density gradient (first derivative of the electron density) and the sign of the Laplacian (second derivative of the electron density). The plots were created using the VMD software along with the visualisation code supplied along with the Multiwfn code.[37]

An isosurface value of 0.05 is used for the representation, where the isosurface indicates reduced density gradient (RDG), colour coded using the sign of the Laplacian, with deep blue (-ve Laplacian)

indicating strong H-bonding, green (near 0 Laplacian) indicating vdW interactions and red (+ve Laplacian) indicating steric hindrance or an absence of electron density.

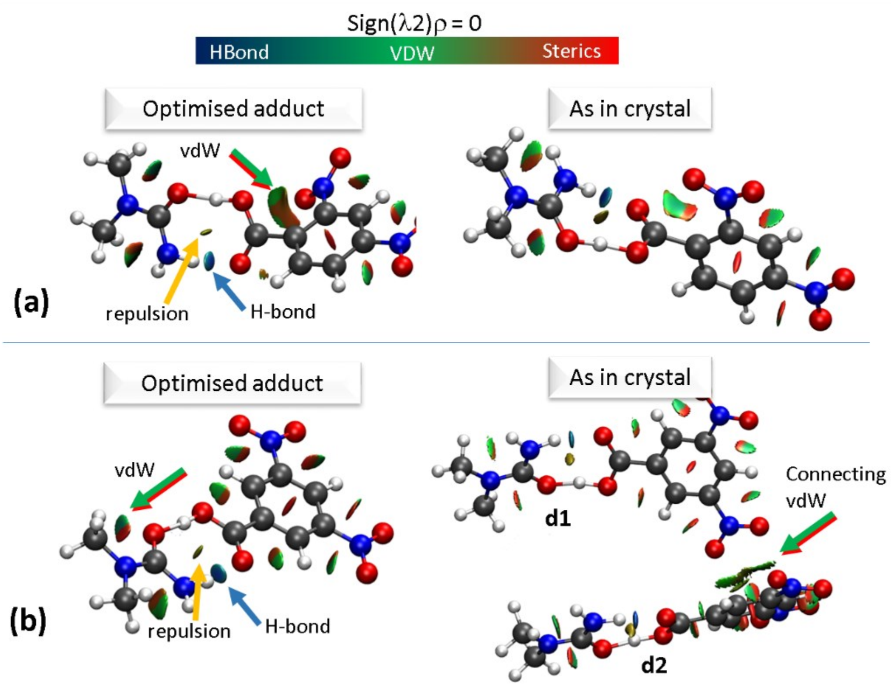

*Figure S7 NCI Isosurface plot for the hydrogen bonded dimer molecular adduct 'as in crystal' in (a) **2** and (b) **3**. The figure was obtained using a reduced density gradient of 2 a.u. and the blue-green-red values ranging from (-0.05 to +0.05 a.u). Green/red arrows point to regions of vdW interactions, while yellow and blue arrows point to mild repulsions inbetween hydrogen bonded rings and strong hydrogen bonds, respectively.*

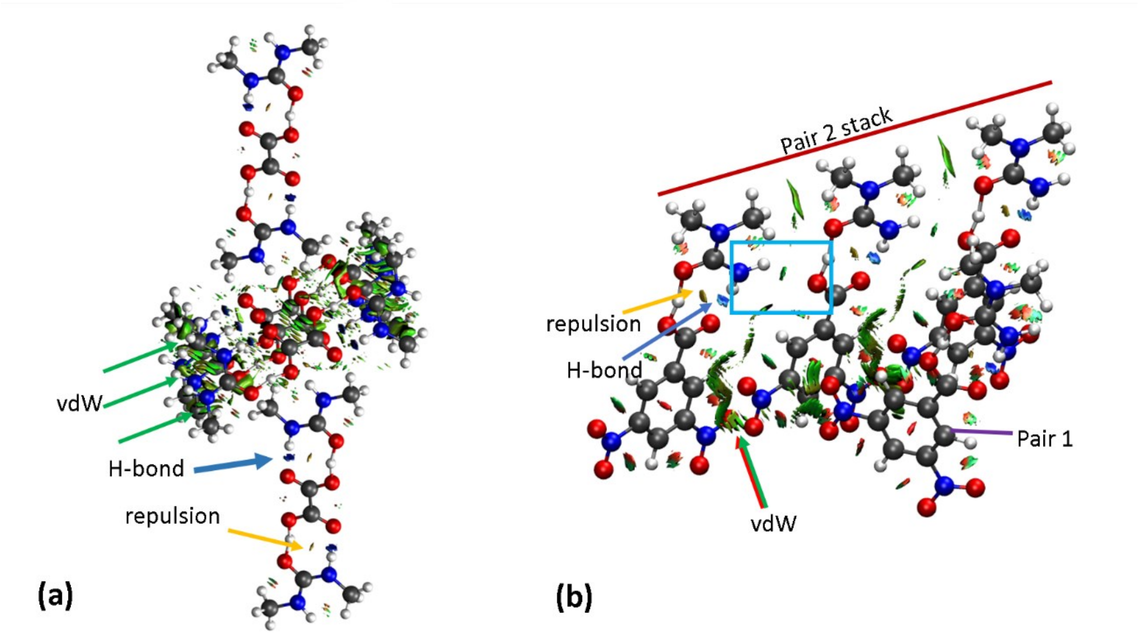

*Figure S8 NCI Isosurface plot for the cluster models (a) **1** and (b) **3d2**. The figure was obtained using a reduced density gradient of 2 a.u. and the blue-green-red values ranging from (-0.05 to +0.05 a.u). Green arrows point to regions of vdW interactions, while yellow and blue arrows point to mild repulsions inbetween hydrogen bonded rings and strong hydrogen bonds, respectively.*

5.4 Electrostatic potentials

Molecular adducts of the hydrogen bonded dimers were optimised in the gas phase using density functional theory (DFT) using Gaussian09[32] code, B3LYP functional and 6-31G+(d,p) basis set.[34] Electrostatic potentials (ESP) and electron densities were also calculated using Gaussian09.[32] Images were generated using the VESTA software package[38] where the electron density is represented and colour based on the ESP. An isosurface of 0.6 was used. Blue regions indicate regions of high electron density and red indicates regions with low electron density.

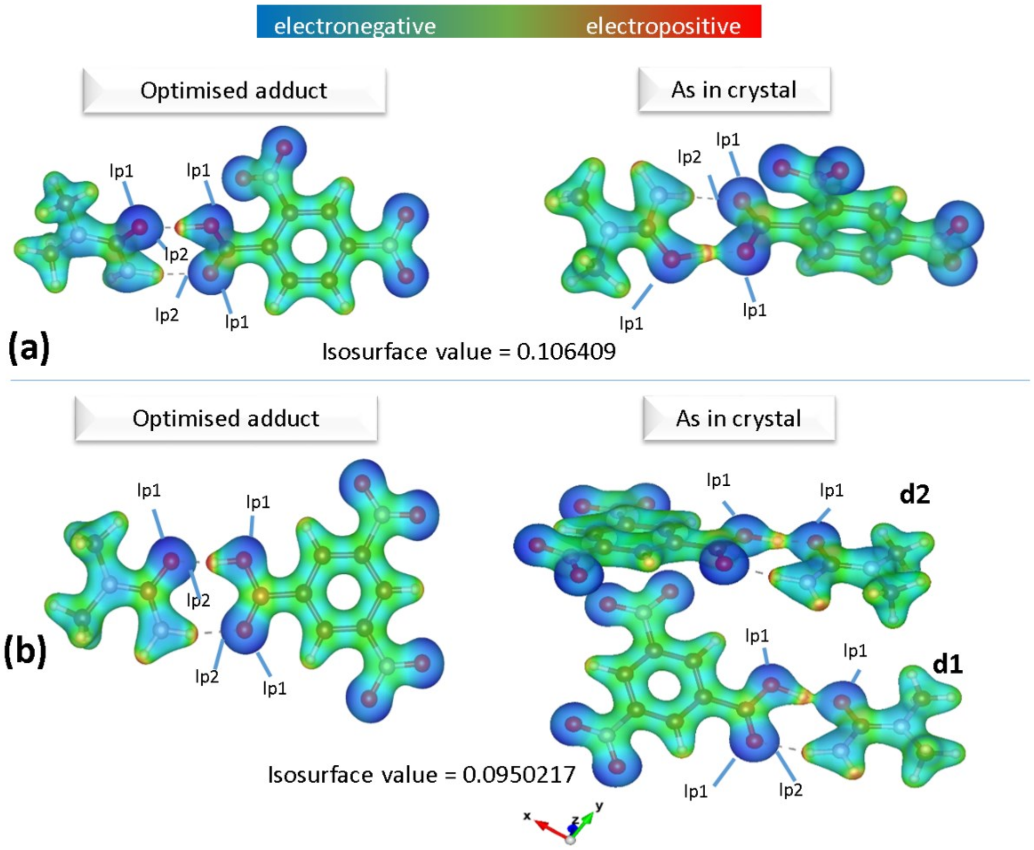

Figure S9 The electrostatic potential surface in (a) ***2*** *and (b)* ***3*** *calculated for the hydrogen bonded dimer molecular adduct 'as in crystal'. Red indicate positive regions, blue negative regions and green van der Waals regions.*

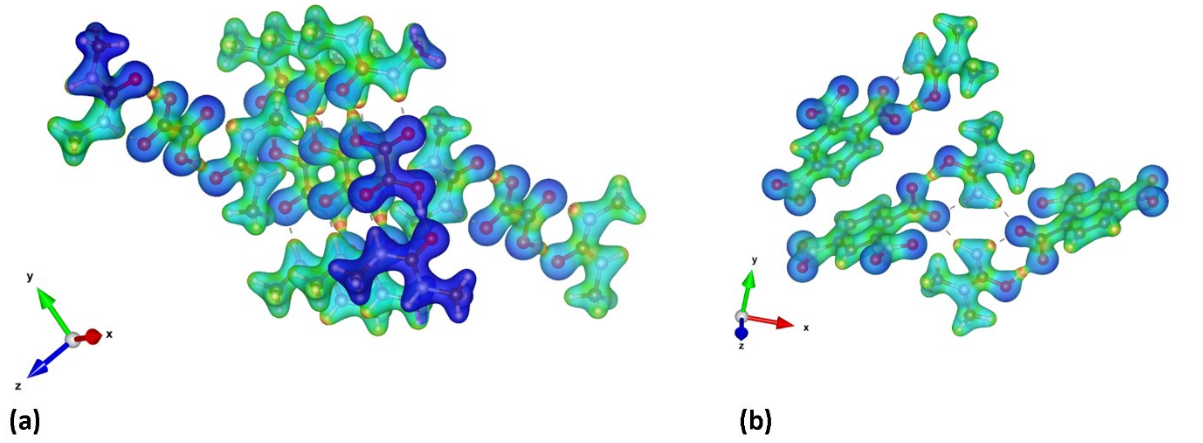

Figure S10 The electrostatic potential surface in (a) ***1*** *and (b)* ***2*** *calculated for the hydrogen bonded dimer in a cluster including nearest neighbours. Red indicate positive regions, blue negative regions and green van der Waals regions.*

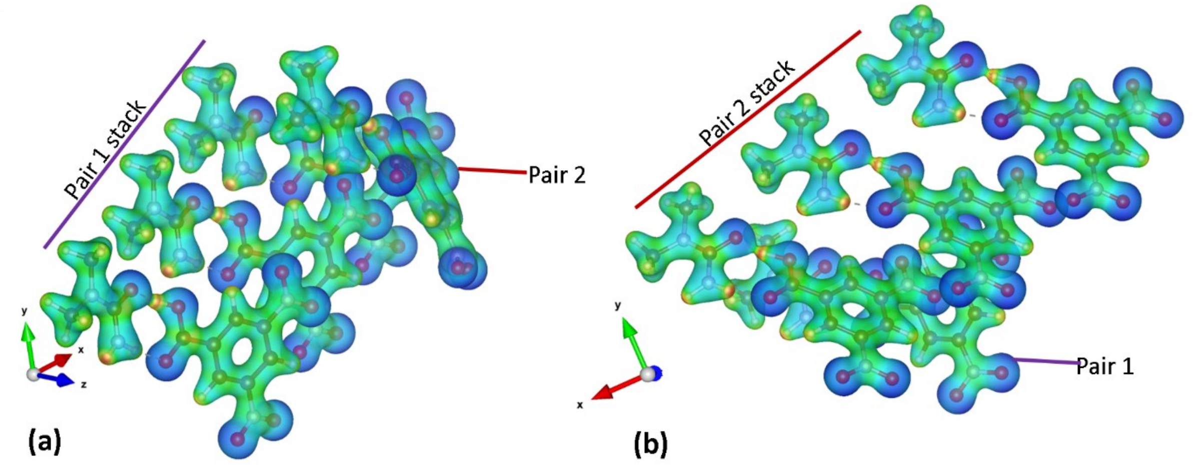

Figure S11 The electrostatic potential surface in (a) ***3d1*** *and (b)* ***3d2*** *calculated for each hydrogen bonded dimer in a cluster including nearest neighbours. Red indicate positive regions, blue negative regions and green van der Waals regions.*

5.5 CLP-PIXEL

CLP-PIXEL[39-41] calculations were performed for **1** – **3** on the hydrogen bonded dimer molecular adducts 'as in crystal'. CIFs were first converted to the correct format using OSCAIL.[42] For **3**, where Z' > 2, individual CIFs and calculations were performed for each molecular pair. Input files (.inp/.gjf) for Gaussian09[32] were prepared using CLP-PIXEL.[39-41] Gaussian09[32] was used to generate the molecular electron density files at the MP2 level with the 6-31G** basis set and default settings. The resulting electron density files were then used as input to the PixelC module of the CLP-PIXEL[39-41] programme package to calculate the total dimer interaction energies.

5.6 H-atom potentials

H-atom potential energy surface (PES) calculations for the H^+ position were carried out on the hydrogen bonded dimer molecular adducts of **1** – **3**, 'as in crystal' and on the optimised structures. H^+ positions were manually changed by 0.02 Å to generate 51 starting structures. Single point energy calculations were carried out on these structures, using Gaussian09[32] code and B3LYP functional and 6-31G+(d,p) basis set.

Similar PES calculations were also carried out using starting structures extracted from temperature-dependent single crystal structures modelled from X-ray and neutron diffraction data, to investigate the effect of temperature on the PES. The starting structures were CIFs reported in the publications by Jones et al (2012)[43] and Saunders et al (2019).[1]

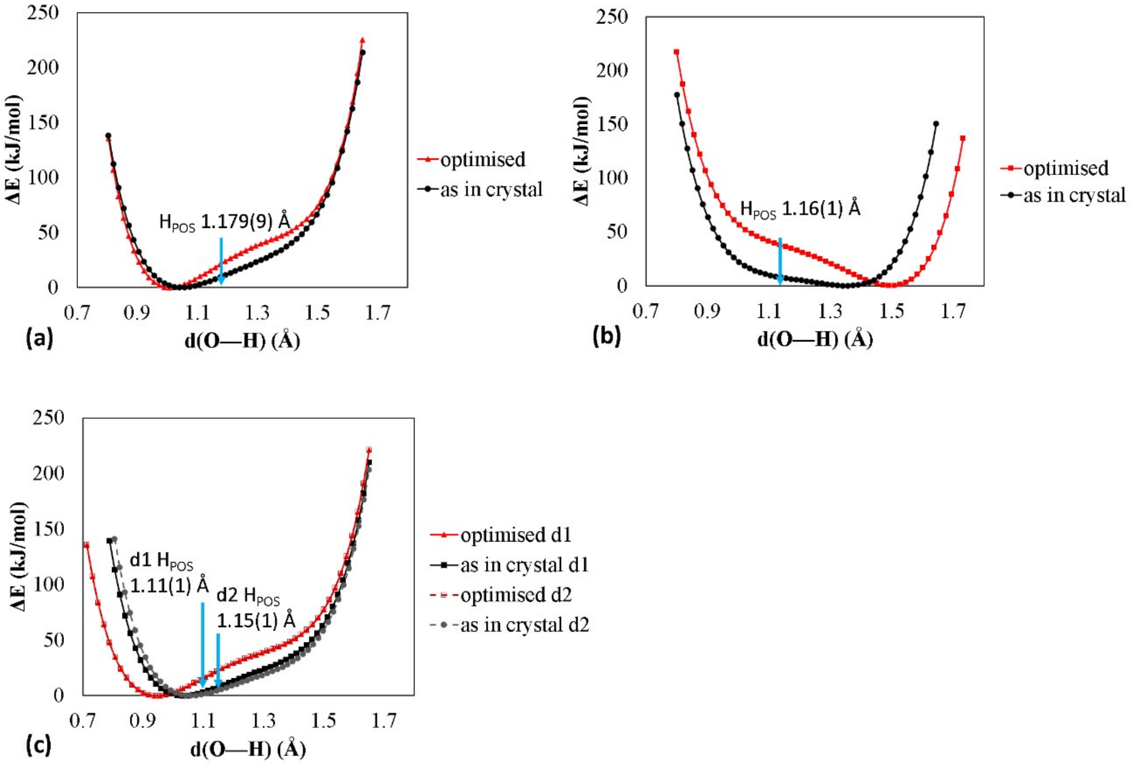

Figure S12 The potential energy surfaces (PES) for the H-atom motion in the short hydrogen bond for (a) ***1****, (b)* ***2*** *and (c)* ***3****. The PES is generated following a manual movement of the hydrogen bond proton by 0.02 Å increments for both the molecular adduct 'as in crystal' and for its optimisation in-vacuo. The potential is plotted in terms of the distance of H from the protonated oxygen atom (x-axis). The H-atom position as found in the crystal structure is indicated by blue arrows (H_{POS}). In the case of* ***2****, the energetic minima favour the acid oxygen acceptor rather than the urea oxygen donor.*

Table S8 The H-atom position in the crystal structure (in terms of O—H atomic separation) versus the H-atom potential energy minima positions for the as in crystal and optimised models in ***1 – 3****.*

System	Crystal structure dO—H (Å)	'As in crystal' minimum dO—H (Å)	Optimised minimum dO—H (Å)
**1**	1.18(1)	1.04	1.01
**2**	1.16(1)	1.36 (H···O_{acid} 1.09)	1.48 (H···O_{acid} 0.96)
**3d1**	1.11(1)	1.03	0.94
**3d2**	1.15(1)	1.06	0.94

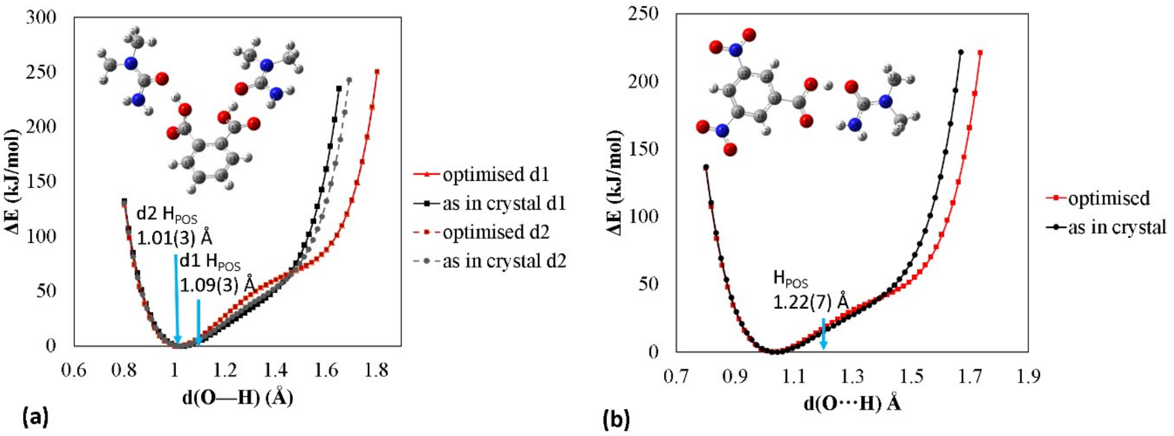

*Figure S13 Short HB dimer fragment optimised in-vacuo (top) and potential energy surfaces following a manual movement of the HB proton by 0.1 Å increments (bottom) for both the molecular adduct 'as in crystal' and for its optimisation in-vacuo for additional literature systems with short O—H···O hydrogen bonds in a $R_2^2(8)$ carboxylic acid:amide hydrogen bonded dimer. (a) N,N-dimethylurea phthalic acid 2:1 (**4**) dO···O 2.452(2), 2.493(2) Å and (b) N,N-dimethylurea 3,5-dinitrobenzoic acid 1:1 (**5**) dO···O 2.445(1) Å. The potential is plotted in terms of the distance of H from the protonated oxygen atom (x-axis). Attempts were made to additionally collect high resolution data for these systems, but the crystals did not diffract to sufficiently high resolution. The H-atom position as found in the crystal structure is indicated by blue arrows (H_{POS}).*

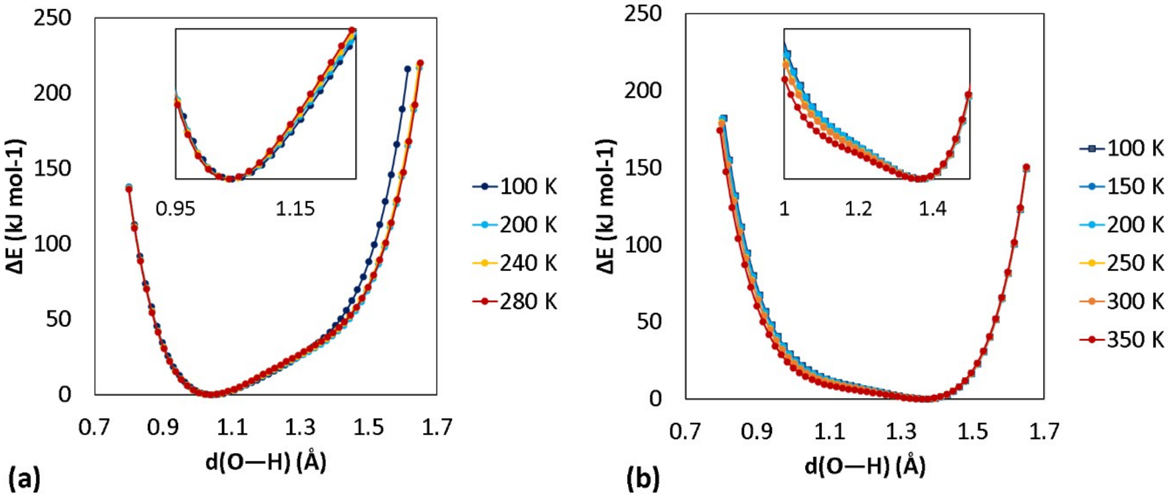

*Figure S14 Potential Energy Surface at each temperature point along the migration pathway for (a) **1** and (b) **2** following a manual movement of the hydrogen bond proton by 0.02 Å increments. The potential is plotted in terms of the distance of H from the protonated oxygen (x-axis).The following CCDC deposited structures have been used: **1** (884859 KIDXEC07 100 K, 884860 KIDXEC06 200 K, 884861 KIDXEC05 240 K, 884862 KIDXEC04 280 K)[43] and **2** (1921988 GUYYIL06 100 K, 1921990 GUYYIL08 150 K, 1921993 GUYYIL11 200 K, 1921989 GUYYIL07 250 K, 1921991 GUYYIL09 300 K, 1921992 GUYYIL10 350K).[1]*

*Table S9 Donor-acceptor distances (dO···O) as a function of temperature in the reported crystal structures for **1** and **2.***

**System 1**			**System 2**		
Temperature (K)	dO···O (Å)	CSD Refcode	Temperature (K)	dO···O (Å)	CSD Refcode
100	2.414(3)	KIDXEC07[43]	100	2.444(1)	GUYYIL06[1]
200	2.448(2)	KIDXEC06[43]	150	2.445(1)	GUYYIL08[1]
240	2.448(2)	KIDXEC05[43]	200	2.448(1)	GUYYIL11[1]
280	2.452(3)	KIDXEC04[43]	250	2.450(1)	GUYYIL07[1]
			300	2.450(1)	GUYYIL09[1]
			350	2.454(2)	GUYYIL10[1]

5.7 Boltzmann distributions

The probability (p) of finding a proton at a given position in the PES is calculated from the partition function Z. By varying T we can predict the change in the probability of finding the proton with temperature. This model however, does not account for the change in the O…O distances with temperature. This could further widen the probability distribution of proton positions.

$$z = \sum_n e^{\frac{-\Delta E_n}{K_b T}}$$

Equation S4

$$p = \frac{1}{z} e^{\frac{-\Delta E}{K_b T}}$$

Equation S5

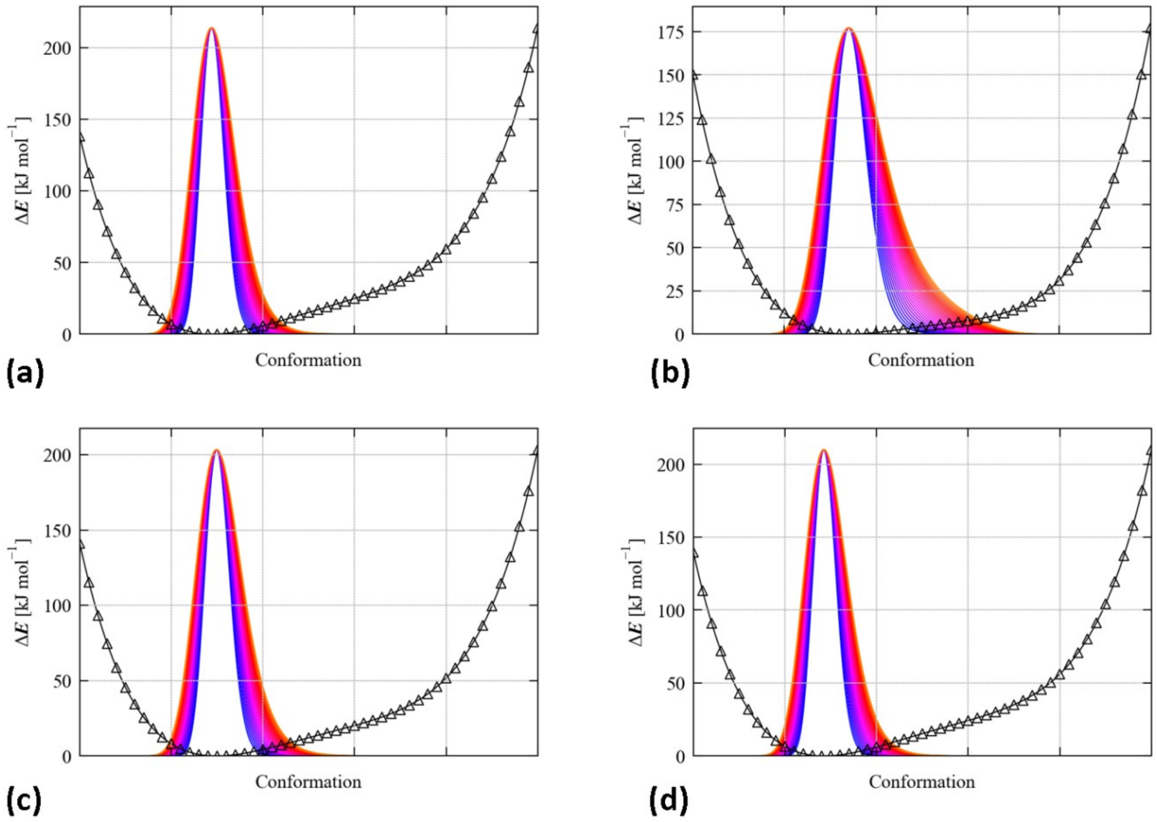

*Figure S15 Calculated Boltzmann distributions for (a) **1**, (b) **2**, (c) **3d1** and (d) **3d2**, calculated with respect to temperature (Blue 100K to 350K Red-orange).*

6. References

1. L. K. Saunders, H. Nowell, L. E. Hatcher, H. J. Shepherd, S. J. Teat, D. R. Allan, P. R. Raithby and C. C. Wilson, *CrystEngComm*, 2019, **21**, 5249-5260.
2. D. Allan, H. Nowell, S. Barnett, M. Warren, A. Wilcox, J. Christensen, L. Saunders, A. Peach, M. Hooper, L. Zaja, S. Patel, L. Cahill, R. Marshall, S. Trimnell, A. Foster, T. Bates, S. Lay, M. Williams, P. Hathaway, G. Winter, M. Gerstel and R. Wooley, *Crystals*, 2017, **7**, 336.
3. E. P. Gibbons, M. T. Heron and N. P. Rees, *13th International Conference on Accelerator and Large Experimental Physics Control Systems (ICALEPCS 2011),10–14 October 2011, Grenoble, France.*, 2012.
4. *DIALS*, Diamond Light Source, Harwell, U.K.
5. R. H. Blessing, *Crystallogr. Rev.*, 1987, **1**.
6. R. Blessing, *J. Appl. Crystallogr.*, 1989, **22**, 396-397.
7. L. Farrugia, *J. Appl. Crystallogr.*, 2012, **45**, 849-854.
8. R. H. Blessing, *Acta Crystallogr. Sect. A*, 1995, **51**, 33-38.
9. D. A. Keen, M. J. Gutmann and C. C. Wilson, *J. Appl. Crystallogr.*, 2006, **39**, 714-722.
10. M. J. Gutmann, *SXD2001*, ISIS Facility, Rutherford Appleton Laboratory, Oxfordshire, England, 2005.
11. G. Sheldrick, *Acta Crystallogr. Sect. A*, 2008, **64**, 112-122.
12. G. Sheldrick, *Acta Crystallogr. Sect. C*, 2015, **71**, 3-8.
13. R. Blessing, *Acta Crystallogr. Sect. B*, 1995, **51**, 816-823.
14. S. C. Capelli, H.-B. Burgi, B. Dittrich, S. Grabowsky and D. Jayatilaka, *IUCrJ*, 2014, **1**, 361-379.
15. O. V. Dolomanov, L. J. Bourhis, R. J. Gildea, J. A. K. Howard and H. Puschmann, *J. Appl. Crystallogr.*, 2009, **42**, 339-341.
16. F. Kleemiss, O. V. Dolomanov, M. Bodensteiner, N. Peyerimhoff, L. Midgley, L. J. Bourhis, A. Genoni, L. A. Malaspina, D. Jayatilaka, J. L. Spencer, F. White, B. Grundkötter-Stock, S. Steinhauer, D. Lentz, H. Puschmann and S. Grabowsky, *Chem. Sci.*, 2021.
17. F. Neese, F. Wennmohs, U. Becker and C. Riplinger, *J. Chem. Phys.*, 2020, **152**, 224108.
18. N. K. Hansen and P. Coppens, *Acta Crystallogr. Sect. A*, 1978, **34**, 909-921.
19. A. Volkov, P. Macchi, L. J. Farrugia, C. Gatti, P. Mallinson, T. Richter and T. Koritsanszky, *XD2006 - a computer program for multipole refinement, topological analysis of charge densities and evaluation of intermolecular energies from experimental or theoretical structure factors.*, 2006.
20. B. Dittrich, C. B. Hubschle, K. Propper, F. Dietrich, T. Stolper and J. J. Holstein, *Acta Crystallogr. Sect. B*, 2013, **69**, 91-104.
21. C. B. Hubschle, P. Luger and B. Dittrich, *J. Appl. Crystallogr.*, 2007, **40**, 623-627.
22. R. Herbst-Irmer and D. Stalke, *Acta Crystallogr. Sect. B*, 2017, **73**, 531-543.
23. R. Herbst-Irmer, J. Henn, J. J. Holstein, C. B. Hübschle, B. Dittrich, D. Stern, D. Kratzert and D. Stalke, *J. Phys. Chem. A*, 2013, **117**, 633-641.
24. C. B. Hubschle and B. Dittrich, *J. Appl. Crystallogr.*, 2011, **44**, 238-240.
25. K. Meindl and J. Henn, *Acta Crystallogr. Sect. A*, 2008, **64**, 404-418.
26. V. V. Zhurov, E. A. Zhurova and A. A. Pinkerton, *J. Appl. Crystallogr.*, 2008, **41**, 340-349.
27. V. Zavodnik, A. Stash, V. Tsirelson, R. de Vries and D. Feil, *Acta Crystallogr. Sect. B*, 1999, **55**, 45-54.
28. A. O. Madsen and A. A. Hoser, *J. Appl. Crystallogr.*, 2014, **47**, 2100-2104.
29. Y. Abramov, *Acta Crystallogr. Sect. A*, 1997, **53**, 264-272.
30. I. Majerz and M. J. Gutmann, *RSC Adv.*, 2015, **5**, 95576-95584.
31. G. K. H. Madsen, B. B. Iversen, F. K. Larsen, M. Kapon, G. M. Reisner and F. H. Herbstein, *J. Am. Chem. Soc.*, 1998, **120**, 10040-10045.
32. G. W. T. M. J. Frisch, H. B. Schlegel, G. E. Scuseria, M. A. Robb, J. R. Cheeseman, G. Scalmani, V. Barone, B. Mennucci, G. A. Petersson, H. Nakatsuji, M. Caricato, X. Li, H. P. Hratchian, A. F. Izmaylov, J. Bloino, G. Zheng, J. L. Sonnenberg, M. Hada, M. Ehara, K. Toyota, R. Fukuda, J.

Hasegawa, M. Ishida, T. Nakajima, Y. Honda, O. Kitao, H. Nakai, T. Vreven, J. A. Montgomery Jr., J. E. Peralta, F. Ogliaro, M. Bearpark, J. J. Heyd, E. Brothers, K. N. Kudin, V. N. Staroverov, R. Kobayashi, J. Normand, K. Raghavachari, A. Rendell, J. C. Burant, S. S. Iyengar, J. Tomasi, M. Cossi, N. Rega, J. M. Millam, M. Klene, J. E. Knox, J. B. Cross, V. Bakken, C. Adamo, J. Jaramillo, R. Gomperts, R. E. Stratmann, O. Yazyev, A. J. Austin, R. Cammi, C. Pomelli, J. W. Ochterski, R. L. Martin, K. Morokuma, V. G. Zakrzewski, G. A. Voth, P. Salvador, J. J. Dannenberg, S. Dapprich, A. D. Daniels, Ö. Farkas, J. B. Foresman, J. V. Ortiz, J. Cioslowski and D. J. Fox, *Gaussian Inc.*, 2009, Wallingford, USA.
33. S. Grimme, *J. Comput. Chem.*, 2006, **27**, 1787-1799.
34. R. Ditchfield, W. J. Hehre and J. A. Pople, *J. Chem. Phys.*, 1971, **54**, 724-728.
35. M. Rivera, M. Dommett and R. Crespo-Otero, *J. Chem. Theory Comput.*, 2019, **15**, 2504-2516.
36. J. Contreras-García, E. R. Johnson, S. Keinan, R. Chaudret, J.-P. Piquemal, D. N. Beratan and W. Yang, *J. Chem. Theory Comput.*, 2011, **7**, 625-632.
37. T. Lu and F. Chen, *J. Comput. Chem.*, 2012, **33**, 580-592.
38. K. Momma and F. Izumi, *J. Appl. Crystallogr.*, 2008, **41**, 653-658.
39. A. Gavezzotti, *New J. Chem.*, 2011, **35**, 1360-1368.
40. A. Gavezzotti, *J. Phys.Chem. B.*, 2003, **107**, 2344-2353.
41. A. Gavezzotti, *Mol. Phys.*, 2008, **106**, 1473-1485.
42. P. McArdle, *J. Appl. Crystallogr.*, 2017, **50**, 320-326.
43. A. O. F. Jones, M.-H. Lemee-Cailleau, D. M. S. Martins, G. J. McIntyre, I. D. H. Oswald, C. R. Pulham, C. K. Spanswick, L. H. Thomas and C. C. Wilson, *Phys. Chem. Chem. Phys.*, 2012, **14**, 13273-13283.
